# PDI Knockdown Inhibits Seizure Activity in Acute Seizure and Chronic Epilepsy Rat Models via *S*-Nitrosylation-Independent Thiolation on NMDA Receptor

**DOI:** 10.3389/fncel.2018.00438

**Published:** 2018-11-22

**Authors:** A. Ran Jeon, Ji-Eun Kim

**Affiliations:** Department of Anatomy and Neurobiology, Institute of Epilepsy Research, College of Medicine, Hallym University, Chuncheon, South Korea

**Keywords:** epilepsy, nitric oxide, protein disulfide isomerase, redox, siRNA, thiol

## Abstract

Redox modulation and *S*-nitrosylation of cysteine residues are the post-translational modifications of *N*-methyl-D-aspartate receptor (NMDAR) to regulate its functionality. Recently, we have reported that protein disulfide isomerase (PDI) reduces disulfide bond (S-S) to free thiol (-SH) on NMDAR. Since PDI is a modulator of *S*-nitrosylation on various proteins, it is noteworthy whether PDI affects *S*-nitrosylation of NMDAR in acute seizure and chronic epilepsy models. In the present study, we found that acute seizures in response to pilocarpine and spontaneous seizures in chronic epilepsy rats led to the reduction in *S*-nitrosylated thiol (SNO-thiol)-to-total thiol ratio on NMDAR, while they elevated nitric oxide (NO) level and *S*-nitrosylation on NMDAR. *N*-nitro-L-arginine methyl ester (L-NAME, a non-selective NOS inhibitor) did not affect seizure activities in both models, although it decreased SNO-thiol levels on NMDAR. However, PDI knockdown effectively inhibited pilocarpine-induced acute seizures and spontaneous seizures in chronic epilepsy rats, accompanied by increasing the SNO-thiol-to-total thiol ratio on NMDAR due to diminishing the amounts of total thiols on GluN1 and GluN2A. Therefore, these findings indicate that PDI may not be a NO donor or a denitrosylase for NMDAR, and that PDI knockdown may inhibit seizure activity by the *S*-nitrosylation-independent thiolation on NMDAR.

## Introduction

Epilepsy is one of the common neurological disorders showing periodic spontaneous seizure activity with a prevalence of 4 – 10 per 1000 of the population (Sander, 2003). The *N*-methyl-D-aspartate receptor (NMDAR) is one of the major excitatory receptors contributing to seizures generation and epileptogenesis (Lipton and Rosenberg, 1994; Kim et al., 2016, 2017c). Since the presence of cysteine residues on the GluN1 and GluN2A subunits of NMDAR (Aizenman et al., 1989; Sullivan et al., 1994; Choi et al., 2000, 2001), redox modulation of disulfide bond (S-S) and *S*-nitrosylation (-SNO) of free thiols (-SH) by nitric oxide (NO) on these residues regulate NMDAR activity (Lei et al., 1992; Choi et al., 2000; Lipton et al., 2002). Therefore, the modulation of NMDAR redox or its *S*-nitrosylation is one of the potential therapeutic targets for epilepsy.

Recently, we have reported that protein disulfide isomerase (PDI) knockdown and its neutralization decrease seizure susceptibility in response to pilocarpine and spontaneous seizure activity in chronic epileptic rats via inhibiting sulfhydration (reduction) of disulfide bonds on NMDAR (Kim et al., 2017c). PDI is a member of the thioredoxin superfamily of redox proteins, which plays a role in catalyzing disulfide bond formation, reduction, and isomerization (Rigobello et al., 2001; Turano et al., 2002; Popescu et al., 2010; Bi et al., 2011). PDI is originally a chaperone in the endoplasmic reticulum (ER), but it is also present in the nucleus, cytosol and cell surface (Edman et al., 1985; Koch, 1987; Yoshimori et al., 1990). Furthermore, it is likely that PDI would be involved in *S*-nitrosylation on NMDAR, since SNO-PDI acts as a transporter for NO (Uehara et al., 2006; Kallakunta et al., 2013). However, it has not been explored whether PDI reductase activity or its capacity as a NO donor affects the reduction and *S*-nitrosylation of cysteine residues on NMDAR, which influence seizure activity.

Here, we demonstrate that acute- and spontaneous seizures elevated NO concentration and the amounts of SNO- and total thiols on GluN1 and GluN2A subunits of NMDAR in acute seizure- and epilepsy models, accompanied by the increased PDI-NMDAR bindings. However, the SNO-thiol-to-total thiol ratios on NMDAR subunits in both models were lower those in control animals. PDI knockdown effectively inhibited seizure activities in both animal models with increasing the fraction of the amount of SNO-thiols in total thiols on NMDAR due to diminishing the total thiols levels on GluN1 and GluN2A. However, *N*-nitro-L-arginine methyl ester (L-NAME, a non-selective NOS inhibitor) did not affect seizure activities in both animal models, although it effectively inhibited NO synthesis. Furthermore, SNO-PDI was not relevant to SNO-thiol levels on NMDAR. Therefore, our findings suggest that PDI may not be a NO carrier for NMDAR, and that PDI knockdown may attenuate seizure activity, independent of *S*-nitrosylation on NMDAR.

## Materials and Methods

### Experimental Animals and Chemicals

Male Sprague-Dawley (SD) rats (7 weeks old) were used in the present study. The colony room was maintained at 22 ± 2°C, 55 ± 5% and a 12:12 light/dark cycle with lights, and food and water *ad libitum* throughout the experiments. All experimental protocols described below were approved by the Institutional Animal Care and Use Committee of Hallym University (Chuncheon, Republic of Korea) and all efforts were made to minimize animal suffering. All reagents were obtained from Sigma-Aldrich (St. Louis, MO, United States), except as noted.

### Surgery, Chemical Infusion and PDI Knockdown

Surgery for a brain infusion kit and an electrode implantation was performed according to our previous study (Kim et al., 2017c). Briefly, animals were anesthetized with isoflurane (3% induction, 1.5–2% for surgery and 1.5% maintenance in a 65:35 mixture of N_2_O:O_2_). A brain infusion kit 1 (Alzet, United States) was implanted into the right lateral ventricle (1 mm posterior; 1.5 mm lateral; 3.5 mm depth from bregma) and connected to an osmotic pump (1007D, Alzet, United States; Reservoir volume, 100 μl) containing (1) control siRNA (20 μM), (2) PDI siRNA (20 μM), (3) vehicle (saline), (4) L-NAME (15 μg/μl) or (5) PACMA31 (a selective PDI inhibitor, 15 μg/μl), respectively. An osmotic pump supplied each animal 0.5 μl/h of vehicle, compound or siRNA over 1 week. A 21-nt siRNA sequence targeting PDI corresponding to coding region (5′ →3′) is sense: CUGCAAAACUGAAGGCAGAUU, and antisense: UCUGCCUUCAGUUUUGCAGUU. A non-silencing RNA (5′-UAAGGCUAUGAAGAGAUAC-3′) was used as the control siRNA. The pump was subcutaneously placed subcutaneously in the interscapular region. Some animals were also implanted by a monopolar stainless steel electrode (Plastics One Inc, United States) or a guide-electrode-combo (C313G-MS303/2/SPC, Plastics One, United States) into the left dorsal hippocampus (3.8 mm posterior; 2.0 mm lateral; 2.6 mm depth from bregma). One week after infusion, animals were used for EEG recording, western blot, co-immunoprecipitation or measurements of thiols and *S*-nitrosylation (see below).

### Acute Seizure Model

Acute seizure model was generated by intraperitoneal injection of pilocarpine as described previously (Kim and Kang, 2011). One week after vehicle or siRNA infusion, rats were anesthetized (urethane, 1.5 g/kg i.p.) and placed in a stereotaxic frame. After the removal of an infusion kit, holes were drilled, and the recording electrode (Plastics One Inc.) and the NO sensor (ISO-NOPF200-L10, World Precision Instruments) were implanted into the left and right dorsal hippocampus (3.8 mm posterior; 2.0 mm lateral; 2.6 mm depth from bregma), respectively. The reference electrode was placed in the posterior cranium over the cerebellum. After establishing a stable baseline for at least 30 min, animals were treated with pilocarpine (380 mg/kg i.p.) 20 min after atropine methylbromide (5 mg/kg i.p.). Some animals were given L-NAME (30 mg/kg, i.p.) 30 min prior to PILO injection. EEG signals and NO concentration were recorded with a DAM 80 differential amplifier (0.1–3000 Hz bandpass; World Precision Instruments, United States) and Free radical analyzer (TBR4100, World Precision Instruments, United States). The data were digitized and analyzed using LabChart Pro v7 software (AD Instruments, NSW, Australia). Two hour after seizure on-set, diazepam (Valium; Hoffman la Roche, Neuilly sur-Seine, France; 10 mg/kg, i.p.) was administered and repeated, as needed. Total power and NO concentration were measured during the 270-min recording session from each animal by LabChart Pro v7 (AD Instruments, Australia). Spectrograms were automatically calculated using a Hanning sliding window with 50% overlap. After recording, animals were immediately decapitated, and used for western blot, co-immunoprecipitation or measurements of thiols and *S*-nitrosylation (see below).

### Generation of Chronic Epilepsy Model

Rats were treated with pilocarpine (380 mg/kg i.p.) 20 min after atropine methylbromide (5 mg/kg i.p.). Control animals received an equal volume of normal saline instead of PILO after the pretreatment with atropine methylbromide. Diazepam (Valium; Hoffman la Roche, France; 10 mg/kg, i.p.) was administered 2 h after on-set of status epilepticus (a prolonged seizure activity, SE) and repeated, as needed. Animals were video-monitored 8 h a day for general behavior and occurrence of spontaneous seizures by 6 weeks after SE. Rats showing spontaneous recurrent seizures were used as chronic epilepsy animals (Ko and Kang, 2015; Kim et al., 2017c).

### Analysis of Chronic Seizure Activity

We applied a modified protocol for the effect of PDI knockdown on spontaneous seizure activity in chronic epileptic rats based on Ko and Kang (2015) and Kim et al. (2017c). After baseline seizure activity (control siRNA treatment) was determined over 2 days, PDI siRNA was administered over a 7-day period using an osmotic pump (1007D, Alzet, United States). Between trials, the minipump was changed out under isoflurane anesthesia. Every day, each animal was applied by video-EEG monitoring (2 h/day) at the same time. EEG analysis was performed by LabChart Pro v7 (AD Instruments, Australia). Behavioral seizure severity was evaluated according to Racine’s scale: 1, immobility, eye closure, twitching of vibrissae, sniffing, facial clonus; 2, head nodding associated with more severe facial clonus; 3, clonus of one forelimb; 4, rearing, often accompanied by bilateral forelimb clonus; and 5, rearing with loss of balance and falling accompanied by generalized clonic seizures. After a 9-day recording, rats were used for western blot, co-immunoprecipitation or measurements of thiols and *S*-nitrosylation (see below). For the measurement of EEG and NO level, 1 week after vehicle or siRNA infusion, some chronic epilepsy animals were applied with the same method to the acute seizure model without atropine methylbromide, pilocarpine, and diazepam treatments. Spectrograms were automatically calculated using a Hanning sliding window with 50% overlap. After recording, rats were used for western blot, co-immunoprecipitation or measurements of thiols and *S*-nitrosylation (see below).

### Analysis of Neuronal Activity in Responses to NMDA and AMPA

In control animals, guide-cannula (3260PGA, Plastics One Inc., United States) and monopolar stainless steel electrode (Plastics One Inc., United States) were implanted into the right lateral ventricle and the left dorsal hippocampus, respectively, and connected to an osmotic pump (1003D, Alzet, United States; Reservoir volume, 100 μl) containing control siRNA or PDI siRNA by the same methods aforementioned. An osmotic pump supplied each animal 1 μl/h of siRNA over 3 days. Three days after siRNA infusion, rats were anesthetized (urethane, 1.5 g/kg i.p.) and placed in a stereotaxic frame. After baseline recording for at least 30 min, an internal infusion cannula (C315IA, Plastics One, United States) was inserted into the lumen of the guide cannula to inject NMDA (20 μM) into the ventricle over a 1-min period using a microinjection pump (1 μl/min, KD Scientific, United States). Animals implanted with a guide-electrode-combo (C313G-MS303/2/SPC, Plastics One, United States) aforementioned were directly infused NMDA or α-amino-3-hydroxy-5-methyl-4-isoxazolepropionic acid (AMPA; 20 μM, respectively) into the hippocampus (3.8 mm posterior; 2.0 mm lateral; 2.6 mm depth from bregma) with the same method. EEG signals were digitized and analyzed using LabChart Pro v7 (AD Instruments, Australia). Spectrograms were automatically calculated using a Hanning sliding window with 50% overlap (Kim et al., 2016, 2017b).

### Western Blot

Under urethane anesthesia (1.5 g/kg, i.p.), the hippocampus was dissected out and homogenized in lysis buffer (50 mM Tris containing 50 mM 4-(2-hydroxyethyl)-1-piperazineethanesulfonic acid (pH 7.4), ethylene glycol tetraacetic acid (pH 8.0), 0.2% Tergitol type NP-40, 10 mM ethylenediaminetetraacetic acid (pH 8.0), 15 mM sodium pyrophosphate, 100 mM β-glycerophosphate, 50 mM NaF, 150 mM NaCl, 2 mM sodium orthovanadate, 1 mM phenylmethylsulfonyl fluoride, and 1 mM dithiothreitol). Total protein content was measured by BCA protein assay kit. Western blotting was performed according to standard procedures. The primary antibodies were mouse anti-PDI (1:1,000, Abcam, United Kingdom), rabbit anti-GluN1 (1:1,000, Millipore, United States) and rabbit anti-GluN2A (1:1,000, Thermo Fisher Scientific, United States). Mouse TrueBlot (Rockland, United States) was used as a secondary antibody for PDI in co-immunoprecipitation. The rabbit anti-β-actin primary antibody (1:6000) was used as internal reference. The signals were scanned and quantified on ImageQuant LAS4000 system (GE health, United States). The values of each sample were normalized with the corresponding amount of β-actin.

### Co-immunoprecipitation

The hippocampal tissues were lysed in radioimmune precipitation buffer (RIPA) with protease and phosphatase inhibitor cocktails (Roche Applied Sciences, United States) and 1 mM sodium orthovanadate. After calibration of total protein concentrations, and equal amounts of proteins were precipitated with the primary antibody and subsequent protein G sepharose at 4°C overnight (Kim et al., 2017b). Beads were collected, eluted in sample buffer and boiled at 95°C for 5 min. Next, Western blotting was performed according to standard procedures.

### Measurement of Free- and Nitrosothiols

Modified biotin switch assay was performed with the *S*-nitrosylation Western Blot Kit (Thermo Fisher Scientific, United States) according to the manufacturer’s protocol. Briefly, lysates were reacted with ascorbate in HENS buffer for specific labeling with iodoTMTzero reagents with MMT pretreatment (SNO-thiol) or not (total thiol). Protein labeling can be confirmed by Western blot using TMT antibody. Thereafter, TMT-labeled proteins were purified by Anti-TMT Resin, eluted by TMT elusion buffer, and identified by Western blot according to standard procedures. For technical controls, we omitted ascorbate for each sample. The ratio of TMT-protein to total protein was described as SNO- and total thiol level. In addition, SNO-thiol over each NMDAR subunit ratio to total-thiol over each NMDAR subunit ratio was described as SNO-thiol-to-total thiol ratios.

### Measurement of PDI Activity

The hippocampal tissues were lysed in RIPA buffer, and protein concentrations were determined by BCA protein assay (Pierce, Rockford, IL, United States). Equal amounts of total proteins (1 mg) were used to measure PDI activity with PROTEOSTAT^®^ PDI assay kit (Enzo life sciences, Farmingdale, NY, United States) according to the manufacturer’s protocol.

### Immunohistochemistry

Rats were anesthetized with urethane anesthesia (1.5 g/kg, i.p.) and perfused transcardially with 4% paraformaldehyde in 0.1 M phosphate buffer (PB, pH 7.4). Brains were post-fixed in the same fixative overnight and then cryoprotected and sectioned at 30 μm with a cryostat. Free-floating coronal sections were incubated in PDI antibody in PBS containing 0.3% Triton X-100 overnight at room temperature. Tissue sections were developed in 3,3′-diaminobenzidine in 0.1 M Tris buffer and mounted on gelatin-coated slides. Some sections were incubated with PDI antibody in PBS containing 0.3% Triton X-100 overnight at room temperature. Thereafter, sections were visualized with Cy2-conjugated secondary antibody. Immunoreaction was observed using an Axio Scope microscope (Carl Zeiss Inc., Oberkocken, Germany). To establish the specificity of the immunostaining, a negative control test was carried out with preimmune serum instead of the primary antibody. No immunoreactivity was observed for the negative control in any structures. All experimental procedures in this study were performed under the same conditions and in parallel.

### Statistical Analysis

Quantitative data are expressed as mean ± standard error of the mean. Data are analyzed by Student *t*-test or ANOVA followed by Newman–Keuls *post hoc* test. A *p* < 0.05 is considered to be statistically different.

## Results

### Acute Seizures Induce the Sustained NO Synthesis Independent of Seizure Activity

**FIGURE 1:**
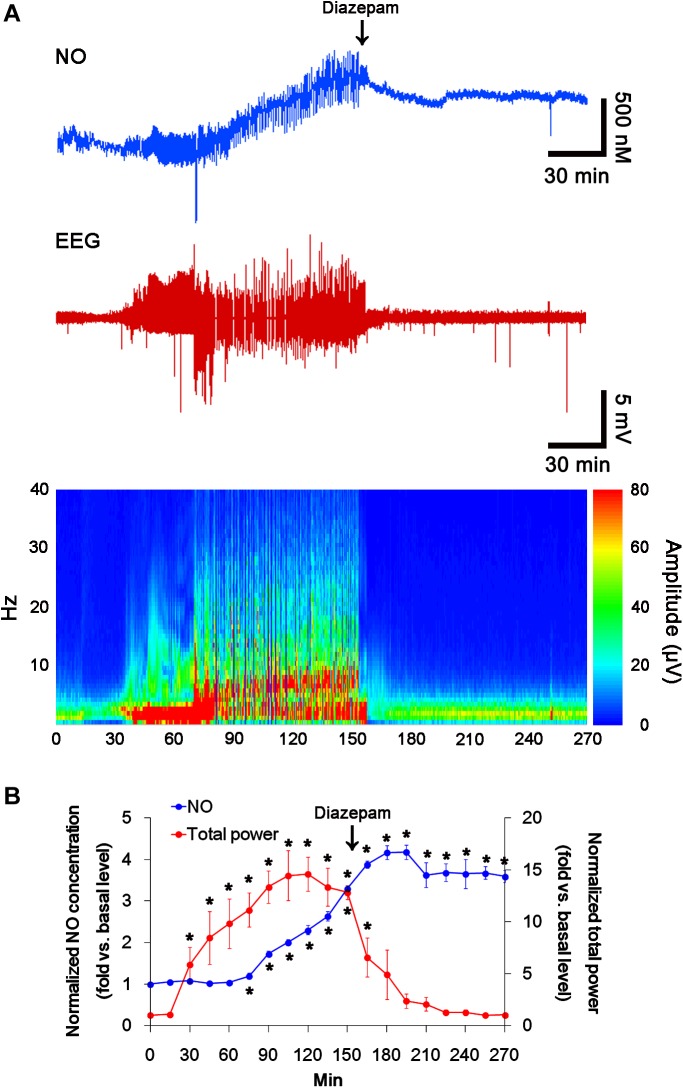
The real-time simultaneous monitoring of NO and EEG after pilocarpine injection. NO level elevates after pilocarpine injection, and gradually increases during SE. Diazepam treatment recovers total EEG power to basal level, but not NO level. **(A)** Representative NO concentration, EEG trace, and frequency-power spectral temporal maps in response to pilocarpine. **(B)** Quantification of NO level and total EEG power in response to PILO (mean ± S.E.M.; *^∗^p* < 0.05 vs. basal level; *n* = 7, respectively).

**FIGURE 2:**
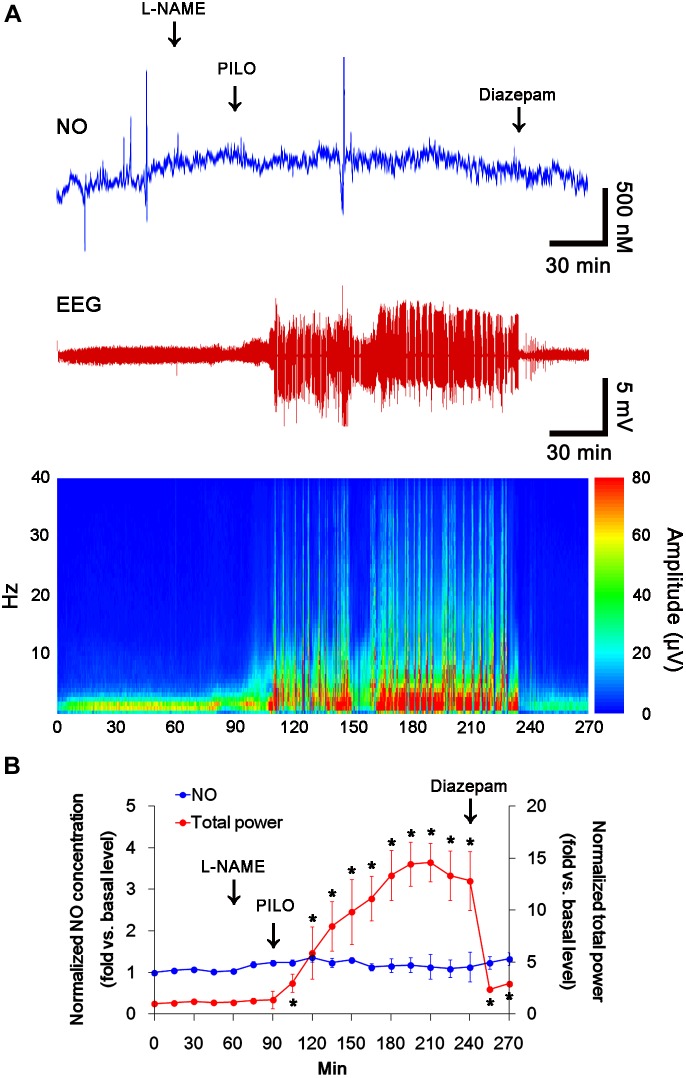
The effect of L-NAME on acute seizure activity in response to pilocarpine. L-NAME effectively inhibits NO synthesis, although it does not affect seizure activity following pilocarpine injection. **(A)** Representative NO concentration, EEG trace and frequency-power spectral temporal maps in response to pilocarpine. **(B)** Quantification of NO level and total EEG power in response to PILO (mean ± S.E.M.; *^∗^p* < 0.05 vs. basal level; *n* = 7, respectively).

In previous studies, we have reported that pilocarpine-induced SE triggers signaling cascades for the NO synthesis, and subsequently increases NO metabolites (Kim et al., 2013; Ko et al., 2015a). Due to the microdialysis method for nitrate/nitrite products in these previous studies, the precise changes in NO synthesis during seizure activity have been limited. In the present study, thus, we applied the real-time simultaneous monitoring of NO and EEG after pilocarpine injection to directly elucidate the relationship between seizure activity and NO generation. EEG revealed seizure on-set and increase in total EEG power ∼30 min after pilocarpine injection (*p* < 0.05 vs. basal level, Figures 1A,B). In addition, NO monitoring showed that NO level increased ∼75 min after pilocarpine injection, and gradually elevated during SE (*p* < 0.05 vs. basal level, Figures 1A,B). After diazepam treatment, total EEG power was recovered to basal level, while increased NO level was sustained (*p* < 0.05 vs. basal level). These findings indicate that seizure on-set may turn on NO generation, but seizure termination may not turn it off. For direct investigating the role of NO in seizure activity, we applied L-NAME (a non-selective NOS inhibitor) to animals 30 min prior to SE induction. L-NAME prevented the prolonged NO generation without affecting seizure activity in response to pilocarpine (Figures 2A,B). Vehicle did not influence seizure susceptibility and its severity (data not shown). These findings indicate that NO may not have pro-convulsive or anti-convulsive effect. Therefore, our findings suggest that prolonged NO generation may be involved in the diverse post-SE events independent of seizure activity.

### Acute Seizure Activity Increases the Amount of Total Thiol and *S*-Nitrosylation on NMDAR

Free thiols on GluN1 and GluN2A subunits undergo *S*-nitrosylation and further oxidation to disulfide bonds, which decreases NMDAR functionality (Jaffrey et al., 2001; Lipton et al., 2002). Therefore, it is likely that NO generation would be one of adaptive responses to SE for reducing seizure activity by nitrosylating free thiols on NMDAR subunits and PDI. To confirm this hypothesis, we measured the amounts of total (SH- + SNO-) thiols and SNO-thiols on NMDAR and PDI.

**FIGURE 3:**
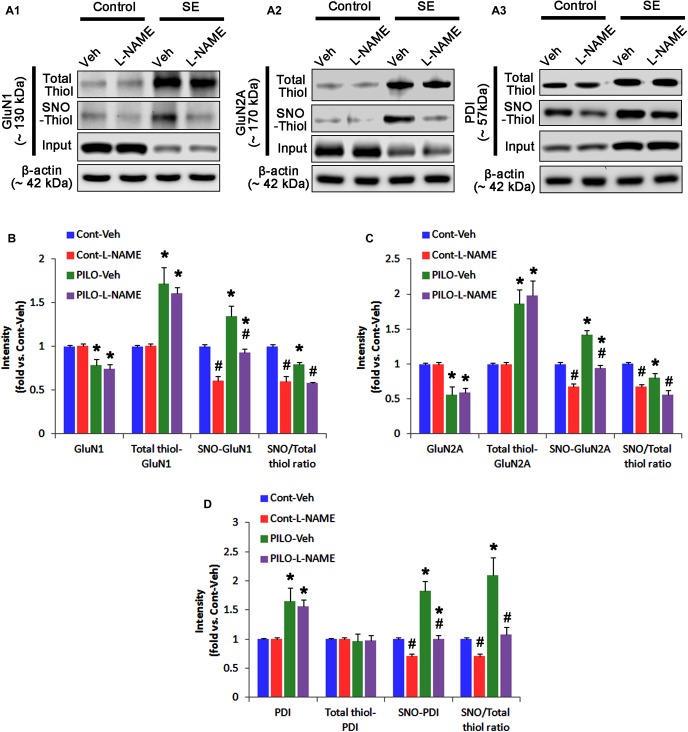
The effect of L-NAME on SNO- and total thiol levels on NMDAR subunits and PDI in the acute seizure model. **(A)** Representative western blot for expressions, and the amounts of total- and SNO-thiol on GluN1 **(A1)**, GluN2A **(A2)**, and PDI **(A3)**. Acute seizure activity increases total- and SNO-thiol levels, but reduce the SNO-thiol-to-total thiol ratio on NMDAR. L-NAME reduces SNO-thiol level and the SNO-thiol-to-total thiol ratios on both NMDAR subunits and PDI without changing their total thiol levels. **(B–D)** Quantification of expressions (panel 1), and the amounts of total thiols (panel 2), SNO-thiol (panel 3) and the SNO-thiol-to-total thiol ratio (SNO ratio; panel 4) on GluN1 **(B)**, GluN2A **(C)**, and PDI **(D)**. Error bars indicate SEM (*^∗^,^#^p* < 0.05 vs. control and vehicle, respectively; *n* = 7, respectively).

In control animals, L-NAME did not affect the expression levels of GluN1 and GluN2A (Figures 3A–C and Supplementary Figure 4), while it reduced the amounts of SNO-thiols, but not total thiols, on both NMDAR subunits (*p* < 0.05 vs. vehicle; Figures 3A–C). The SNO-thiol-to-total thiol ratios on GluN1 and GluN2A were decreased to 0.6- and 0.68-fold of vehicle level, respectively (*p* < 0.05; Figures 3B,C). L-NAME did not influence PDI expression level and the amount of total thiols on PDI. Since *S*-nitrosylation level of PDI was decreased by L-NAME (Figures 3A3,D), the SNO-thiol-to-total thiol ratio on PDI was diminished to 0.71-fold of vehicle level (*p* < 0.05; Figure 3D).

**FIGURE 4:**
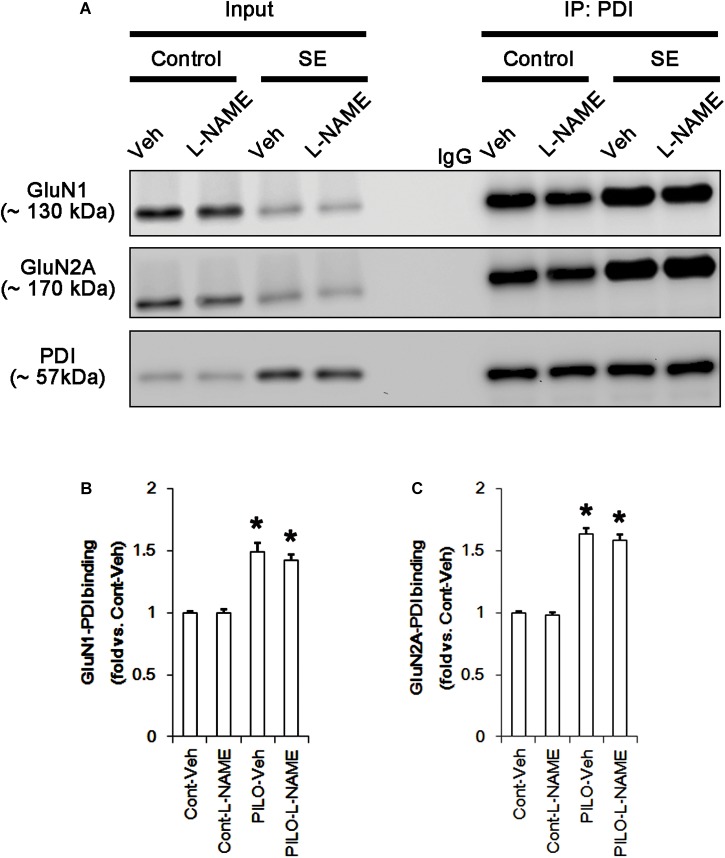
The effect of L-NAME on on the binding of PDI to NMDAR subunits in the acute seizure model. **(A)** Co-immunoprecipitation of PDI and NMDAR. Seizure activity increases PDI-NMDAR bindings that are unaffected by L-NAME. **(B,C)** Quantitative analyses of co-immunoprecipitation of PDI with GluN1 and GluN2A. Error bars indicate SEM (*^∗^p* < 0.05 vs. control; *n* = 7, respectively).

Following acute seizures, the expression levels of GluN1 and GluN2A were reduced to 0.78- and 0.56-fold of control level, respectively (*p* < 0.05; Figures 3A–C). However, the amounts of total- and SNO-thiols on GluN1 were increased to 1.72- and 1.35-fold of control level, respectively (*p* < 0.05; Figures 3A1,B). Thus, the SNO-thiol-to-total thiol ratio on GluN1 was decreased to 0.79-fold of control level (*p* < 0.05; Figure 3B). Similarly, acute seizures increased the amount of total thiols and *S*-nitrosylation on GluN2A (*p* < 0.05; Figures 3A2,C), while the SNO-thiol-to-total thiol ratio on GluN2A was abolished to 0.8-fold of control level (*p* < 0.05; Figure 3C). Consistent with our previous studies (Ko et al., 2015b; Kim et al., 2017c), acute seizures elevated PDI expression and its *S*-nitrosylation level without altering the amount of total thiols (*p* < 0.05; Figures 3A3,D). Unlike NMDAR subunits, the SNO-thiol-to-total thiol ratio on PDI was 2.1-fold of control level (*p* < 0.05; Figure 3D). In addition, the bindings of PDI to GluN1 and GluN2A were significantly increased to 1.48- and 1.63-fold of control level, respectively (*p* < 0.05; Figures 4A–C and Supplementary Figure 5). L-NAME abolished *S*-nitrosylation levels of GluN1, GluN2A, and PDI without changing their expression levels following acute seizures (*p* < 0.05 vs. vehicle; Figures 3A–D). L-NAME could not affect the amount of total thiols on NMDAR subunits and PDI (Figures 3A–D). The SNO-thiol-to-total thiol ratios on GluN1, GluN2A and PDI were 0.58-, 0.56-, and 1.08-fold of vehicle level in control animals, respectively (*p* < 0.05; Figures 3B–D). As compared to vehicle, L-NAME did not influence the bindings of PDI to GluN1 and GluN2A under control and post-seizure conditions (*p* < 0.05; Figures 4A–C). Taken together, our findings indicate that seizure-induced NO generation may increase *S*-nitrosylation levels of NMDAR subunits and PDI without affecting their disulfide bond formations. Furthermore, the binding of PDI to NMDAR subunits may be unaffected by *S*-nitrosylation of PDI.

### PDI siRNA Inhibits Acute Seizures in Response to Pilocarpine With Reducing the Amount of Total Thiol on NMDAR

In the present study, acute seizure activity increased PDI expression and the amounts of SNO-thiol and total thiol residues on NMDAR subunits, which regulate NMDAR functionality (Aizenman et al., 1989; Tang and Aizenman, 1993; Sanchez et al., 2000; Kim et al., 2017c). Since PDI is a redox enzyme and acts as a NO donor and a denitrosylase (Ramachandran et al., 2001; Rigobello et al., 2001; Turano et al., 2002; Root et al., 2004; Sliskovic et al., 2005; Popescu et al., 2010; Bi et al., 2011; Kallakunta et al., 2013; Kim et al., 2017c), we investigated whether the increased PDI expression affects the redox and *S*-nitrosylation levels on NMDAR following acute seizure activity.

Consistent with our previous studies (Kim et al., 2017c; Lee and Kim, 2018), PDI siRNA effectively reduced PDI expression, its activity and *S*-nitrosylation of dynamin-related protein 1 (DRP1, *p* < 0.05 vs. control siRNA; Supplementary Figures 1, 12). These findings confirm the efficacy of PDI siRNA in the present study. As compared to control siRNA, PDI siRNA did not influence the phosphorylation or expression levels of protein kinase RNA (PKR)-like ER kinase (PERK), inositol-requiring protein 1-α (IRE1α), activating transcription factor 6 (ATF6) and glucose-regulated protein 78 (GRP78), which are involved in the regulation of ER stress (Ko et al., 2015b; Kim et al., 2017a; Supplementary Figures 2, 13). These findings indicate that PDI knockdown may not provoke ER stress under physiological condition.

**FIGURE 5:**
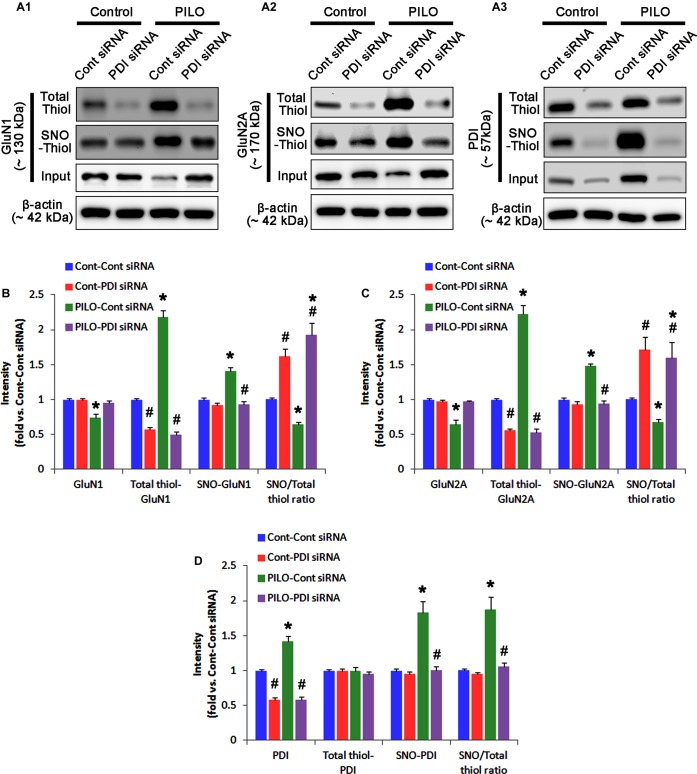
The effect of PDI knockdown on SNO- and total thiol levels on NMDAR subunits and PDI in the acute seizure model. **(A)** Representative western blot for expressions, and the amounts of total- and SNO-thiol on GluN1 **(A1)**, GluN2A **(A2)**, and PDI **(A3)**. PDI siRNA reduces SNO- and total thiol levels on both NMDAR subunits, but increases the SNO-thiol-to-total thiol ratios on both NMDAR subunits under control and post-seizure conditions. **(B–D)** Quantification of expressions (panel 1), and the amounts of total thiols (panel 2), SNO-thiol (panel 3), and the SNO-thiol-to-total thiol ratio (SNO ratio; panel 4) on GluN1 **(B)**, GluN2A **(C)**, and PDI **(D)**. Error bars indicate SEM (*^∗^,^#^p* < 0.05 vs. control and vehicle, respectively; *n* = 7, respectively).

**FIGURE 6:**
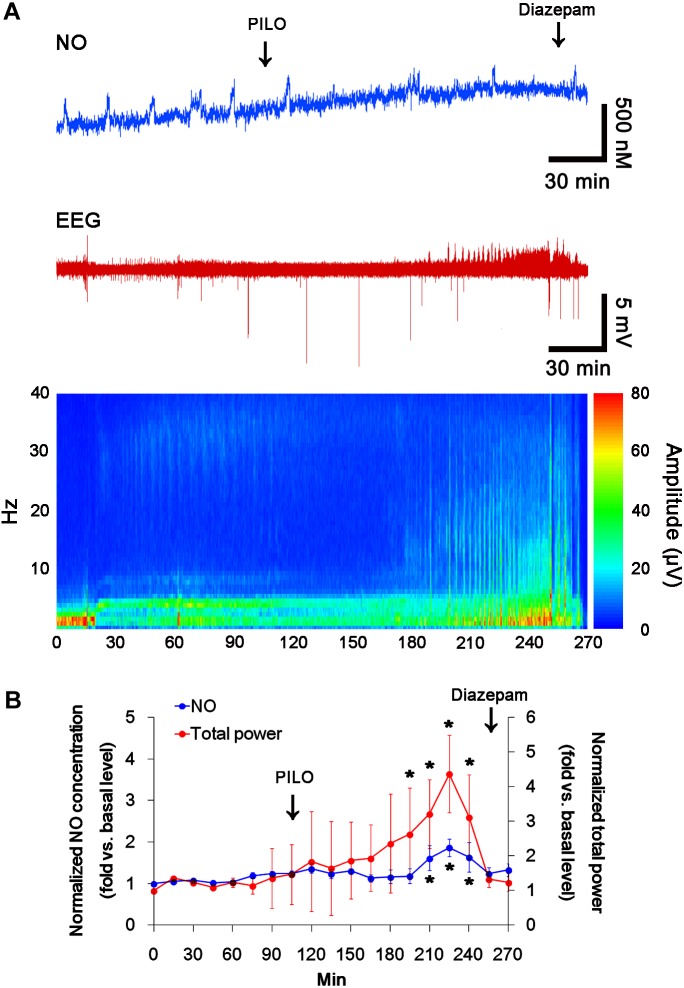
The effect of PDI siRNA on acute seizure activity in response to pilocarpine. PDI knockdown effectively inhibits seizure activity and NO synthesis following pilocarpine injection. **(A)** Representative NO concentration, EEG trace and frequency-power spectral temporal maps in response to pilocarpine. **(B)** Quantification of NO level and total EEG power in response to PILO (mean ± S.E.M.; *^∗^p* < 0.05 vs. basal level; *n* = 7, respectively).

In control animals, PDI knockdown decreased the amount of total thiols on NMDAR subunits (*p* < 0.05 vs. control siRNA; Figures 5A–C and Supplementary Figure 6), while NMDAR subunit expressions and their *S*-nitrosylation were unaffected by PDI siRNA (Figures 5A–C). Thus, the SNO-thiol-to-total thiol ratios on GluN1 and GluN2A were increased to 1.62- and 1.49-fold of control siRNA level, respectively (*p* < 0.05; Figures 5B,C). PDI knockdown delayed seizure on-set and NO generation in response to pilocarpine (*p* < 0.05 vs. control siRNA; Figures 6A,B). However, control siRNA did not affect the seizure susceptibility and its severity in response to pilocarpine (data not shown). PDI knockdown prevented the decreased NMDAR expressions induced by acute seizures (*p* < 0.05 vs. control siRNA; Figures 5A–C). It also abrogated the increases in the amount of total thiols and *S*-nitrosylation on NMDAR following acute seizure activity (*p* < 0.05 vs. control siRNA; Figures 5A–C). Furthermore, PDI knockdown increased the SNO-thiol-to-total thiol ratio on GluN1 and GluN2A to 1.93- and 1.85-fold of vehicle levels in control animals, respectively (*p* < 0.05 vs. control siRNA; Figures 5B,C). Similar to PDI siRNA, PACMA31 (a selective PDI inhibitor) also inhibited seizure activity in response to pilocarpine and diminished the amount of total thiols, but not SNO-thiols, on NMDAR subunits (*p* < 0.05 vs. vehicle; Supplementary Figures 3, 14). Furthermore, PACMA31 increased the SNO-thiol-to-total thiol ratios on GluN1 and GluN2A (*p* < 0.05 vs. vehicle; Supplementary Figures 3, 14). These data suggest that PDI may reduce the disulfide bonds on NMDAR without affecting its *S*-nitrosylation.

**FIGURE 7:**
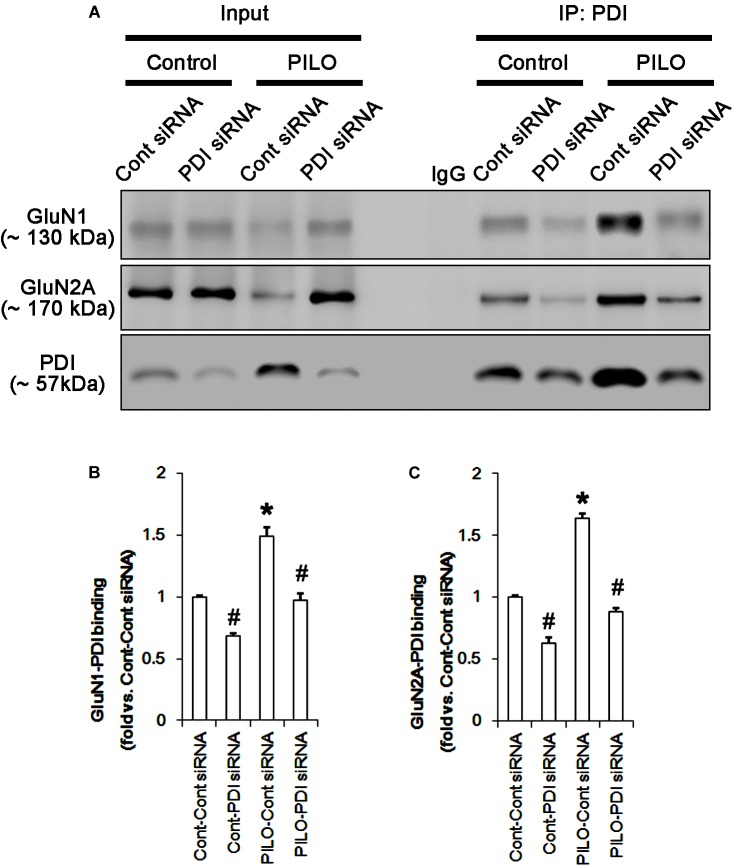
The effect of PDI knockdown on on the binding of PDI to NMDAR subunits in the acute seizure model. **(A)** Co-immunoprecipitation of PDI and NMDAR. PDI knockdown reduces PDI-NMDAR bindings under control and post-seizure conditions. **(B,C)** Quantitative analyses of co-immunoprecipitation of PDI with GluN1 and GluN2A. Error bars indicate SEM (*^∗^,^#^p* < 0.05 vs. control; *n* = 7, respectively).

Protein disulfide isomerase siRNA abolished PDI expression (*p* < 0.05 vs. control siRNA; Figures 5A3,D), but not the amounts of total thiols and SNO-thiols of PDI (Figure 5D). The SNO-thiol-to-total thiol ratio on PDI was unaffected by PDI knockdown (Figure 5D). PDI siRNA inhibited the up-regulation of PDI expression, its *S*-nitrosylation and the SNO-thiol-to-total thiol ratio on PDI induced by acute seizures (*p* < 0.05 vs. control siRNA; Figures 5A3,D). PDI knockdown significantly diminished the bindings of PDI to GluN1 and GluN2A under control and post-seizure conditions (*p* < 0.05 vs. control siRNA; Figures 7A–C and Supplementary Figure 7). Therefore, our findings indicate that PDI knockdown may inhibit seizure activity by increasing the SNO-thiol-to-total thiol ratio on NMDAR due to reducing total thiol level.

### The Amounts of Total Thiol and *S*-Nitrosylation on NMDAR Increase in Chronic Epilepsy Rat

**FIGURE 8:**
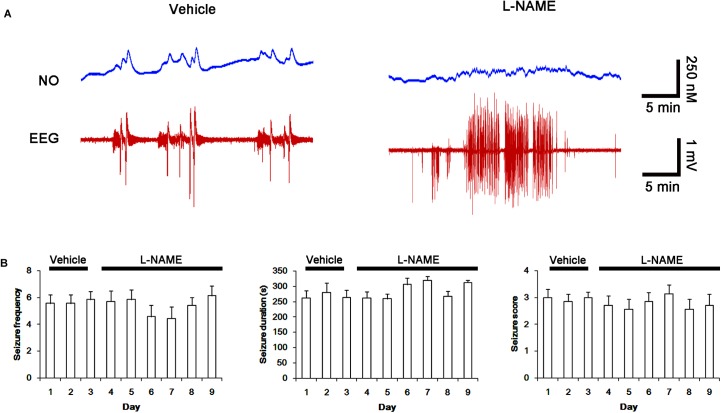
Effect of L-NAME on spontaneous seizure activity and NO generation in chronic epilepsy rats. **(A)** Representative NO concentration and EEG trace for vehicle- and L-NAME-infused epilepsy animals. L-NAME inhibits NO level synthesis, but not spontaneous seizure activity. **(B)** The effect of L-NAME on spontaneous seizure activity: the mean seizure frequency (Left), seizure duration (Middle) and behavioral seizure score (Right). Error bars indicate SEM (*n* = 7, respectively).

Next, we also investigated whether the SNO-thiol-to-total thiol ratio on NMDAR is relevant to spontaneous seizure activity in chronic epileptic animals. In vehicle-infusion period, the mean seizure frequency was ∼5.6/2-h recording session, the total seizure duration was ∼270 s, and behavioral seizure severity (Racine score) was ∼3 (Figures 8A,B). NO level immediately rose after spontaneous seizure on-set, and gradually decreased to basal level after seizure cessation (Figure 8A). L-NAME could not affect seizure frequency, duration and score in chronic epilepsy rat, but effectively inhibited NO synthesis during seizures (Figures 8A,B).

**FIGURE 9:**
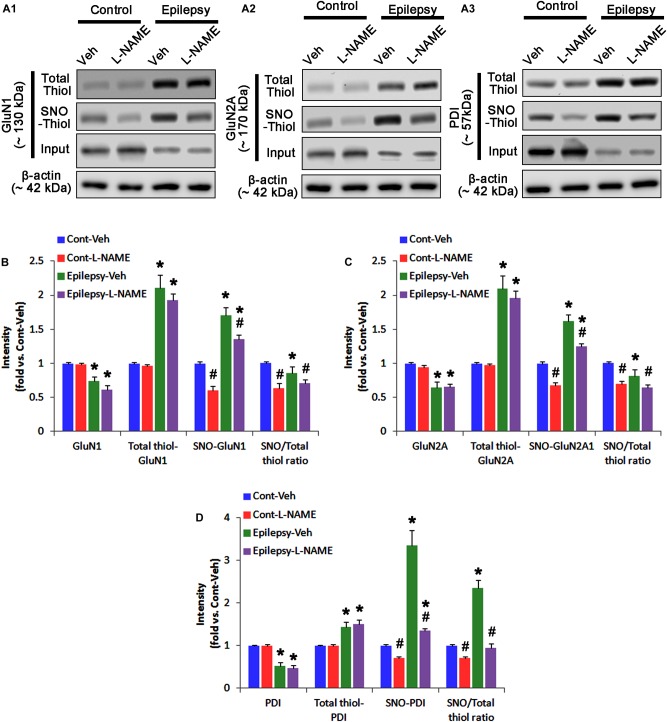
The effect of L-NAME on SNO- and total thiol levels on NMDAR subunits and PDI in chronic epilepsy rats. **(A)** Representative western blot for expressions, and the amounts of total- and SNO-thiols on GluN1 **(A1)**, GluN2A **(A2)**, and PDI **(A3)**. In chronic epilepsy animals, the total- and SNO-thiol levels are increased on NMDAR, but the SNO-thiol-to-total thiol ratios are reduced. L-NAME reduces SNO-thiol level and the SNO-thiol-to-total thiol ratios on both NMDAR subunits and PDI without changing their total thiol levels. **(B–D)** Quantification of expressions (panel 1), the amounts of total thiols (panel 2), SNO-thiol (panel 3), and the SNO-thiol-to-total thiol ratio (SNO ratio; panel 4) on GluN1 **(B)**, GluN2A **(C)**, and PDI **(D)**. Error bars indicate SEM (*^∗^,^#^p* < 0.05 vs. control and vehicle, respectively; *n* = 7, respectively).

**FIGURE 10:**
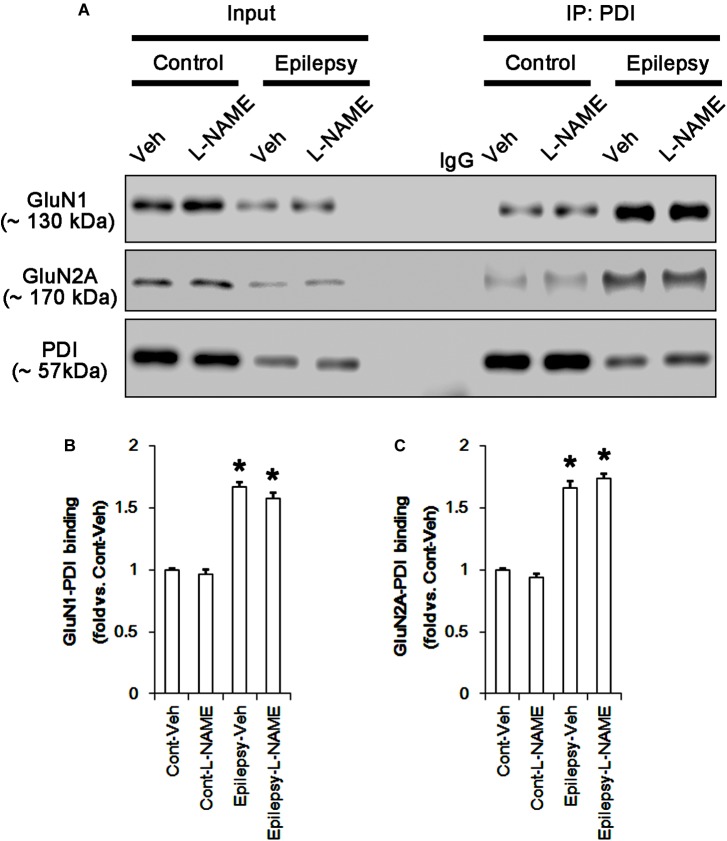
The effect of L-NAME on the binding of PDI to NMDAR subunits in chronic epilepsy model. **(A)** Co-immunoprecipitation of PDI and NMDAR. Chronic epilepsy animals show the increase in PDI-NMDAR bindings. L-NAME cannot affect them. **(B,C)** Quantitative analyses of co-immunoprecipitation of PDI with GluN1 and GluN2A. Error bars indicate SEM (*^∗^p* < 0.05 vs. control; *n* = 7, respectively).

In chronic epilepsy rats, the expression levels of GluN1 and GluN2A were reduced to 0.74- and 0.65-fold of control level, respectively (*p* < 0.05; Figures 9A–C and Supplementary Figure 8). However, the amounts of total and SNO-thiols on GluN1 were increased to 2.1- and 1.71-fold of control level, respectively (*p* < 0.05; Figures 9A1,B). Thus, the SNO-thiol-to-total thiol ratio on GluN1 was decreased to 0.85-fold of control level (*p* < 0.05; Figure 9B). In addition, the amounts of total and SNO-thiol on GluN2A were 2.01- and 1.62-fold of control level, respectively (*p* < 0.05; Figures 9A2,C). The SNO-thiol-to-total thiol ratio on GluN2A was 0.82-fold of control level (*p* < 0.05; Figure 9C). Although PDI expression was 0.55-fold of control level (*p* < 0.05; Figures 9A3,D), the amounts of total and SNO-thiol on PDI were 1.44- and 3.36-fold of control level, respectively (*p* < 0.05; Figures 9A3,D). The SNO-thiol-to-total thiol ratio on PDI was 2.36-fold of control level (*p* < 0.05; Figure 9D). In addition, the bindings of PDI to GluN1 and GluN2A were 1.67- and 1.66-fold of control level, respectively (*p* < 0.05; Figures 10A–C and Supplementary Figure 9). L-NAME abolished *S*-nitrosylation levels of GluN1, GluN2A and PDI to 1.35-, 1.25-, and 1.35-fold of vehicle level in control animals, respectively (*p* < 0.05 vs. vehicle; Figures 9A–D), but not expression levels and the amount of disulfide bonds on NMDAR subunits and PDI (Figures 9A–D). The SNO-thiol-to-total thiol ratios on GluN1, GluN2A and PDI were 0.71-, 0.65-, and 0.95-fold of vehicle level in control animals, respectively (*p* < 0.05; Figures 9B–D). L-NAME did not influence the bindings of PDI to GluN1 and GluN2A in control and epilepsy animals (*p* < 0.05 vs. vehicle; Figures 10A–C). These findings indicate that the decreased SNO-thiol-to-total thiol ratio on NMDAR may be involved in spontaneous seizure activity in chronic epilepsy rats, similar to acute seizures.

### PDI Knockdown Inhibits Spontaneous Seizure Activity With the Increased SNO-Thiol-to-Total Thiol Ratio on NMDAR

**FIGURE 11:**
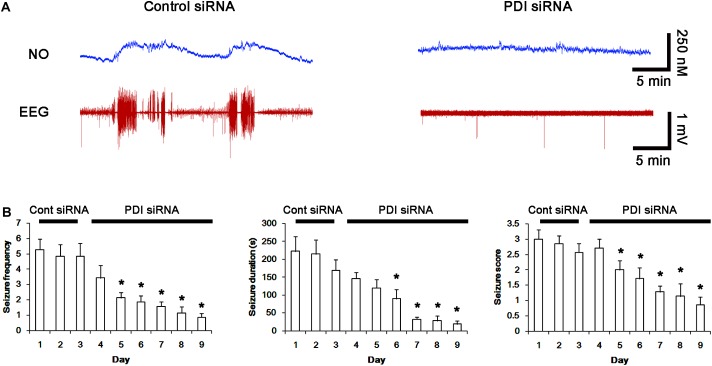
Effect of PDI siRNA on spontaneous seizure activity and NO generation in chronic epilepsy rats. **(A)** Representative NO concentration and EEG trace for control siRNA- and PDI siRNA-infused epilepsy animals. PDI siRNA inhibits spontaneous seizure activity and NO level synthesis. **(B)** The effect of PDI siRNA on spontaneous seizure activity: the mean seizure frequency (Left), seizure duration (Middle) and behavioral seizure score (Right). Error bars indicate SEM (^∗^*p* < 0.05 vs. control siRNA; *n* = 7, respectively).

Next, we investigated the effect of PDI knockdown on NMDAR redox as well as its *S*-nitrosylation in chronic epilepsy rats, because PDI siRNA effectively inhibits the generation of spontaneous seizures in chronic epilepsy rats (Kim et al., 2017c). In control siRNA-infusion period, the mean seizure frequency was ∼5/2-h recording session, the total seizure duration was ∼220 s, and behavioral seizure severity (Racine score) was ∼3 (Figures 11A,B). On the final day in PDI siRNA infusion period (7 day-over PDI siRNA infusion), the mean seizure frequency and the total seizure duration were reduced to ∼0.85/recording session and 19 s, respectively. Behavioral seizure score was also decreased to 1.7 (*p* < 0.05 vs. control siRNA; Figures 11A,B).

**FIGURE 12:**
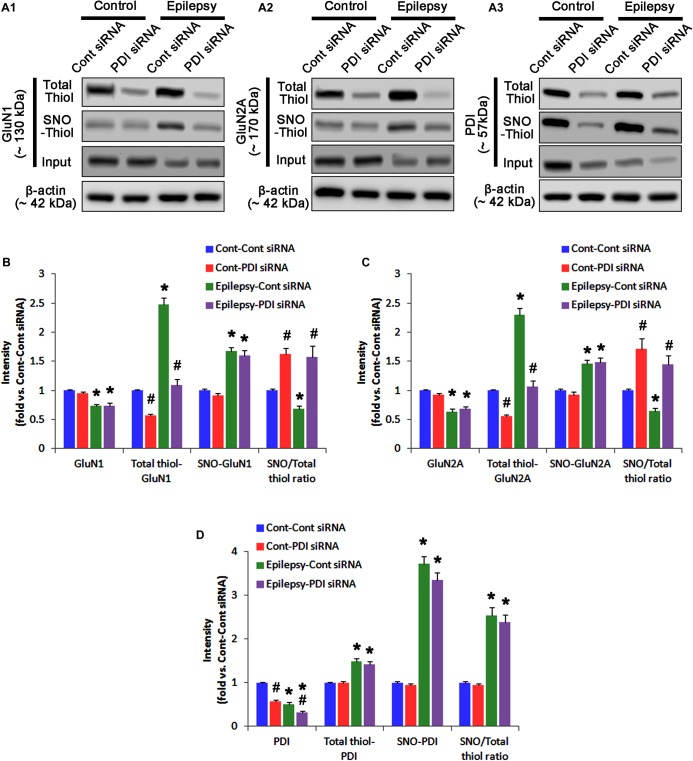
The effect of PDI knockdown on SNO- and total thiol levels on NMDAR subunits and PDI in chronic epilepsy rats. **(A)** Representative western blot for expressions, and the amounts of total- and SNO-thiol on GluN1 **(A1)**, GluN2A **(A2)**, and PDI **(A3)**. PDI siRNA reduces the total thiol level on NMDAR without altering SNO-thiol level. Thus, it increases the SNO-thiol-to-total thiol ratios on both NMDAR subunits. **(B–D)** Quantification of expressions (panel 1), and the amounts of total thiols (panel 2), SNO-thiol (panel 3), and the SNO-thiol-to-total thiol ratio (SNO ratio; panel 4) on GluN1 **(B)**, GluN2A **(C)**, and PDI **(D)**. Error bars indicate SEM (*^∗^,^#^p* < 0.05 vs. control and vehicle, respectively; *n* = 7, respectively).

**FIGURE 13:**
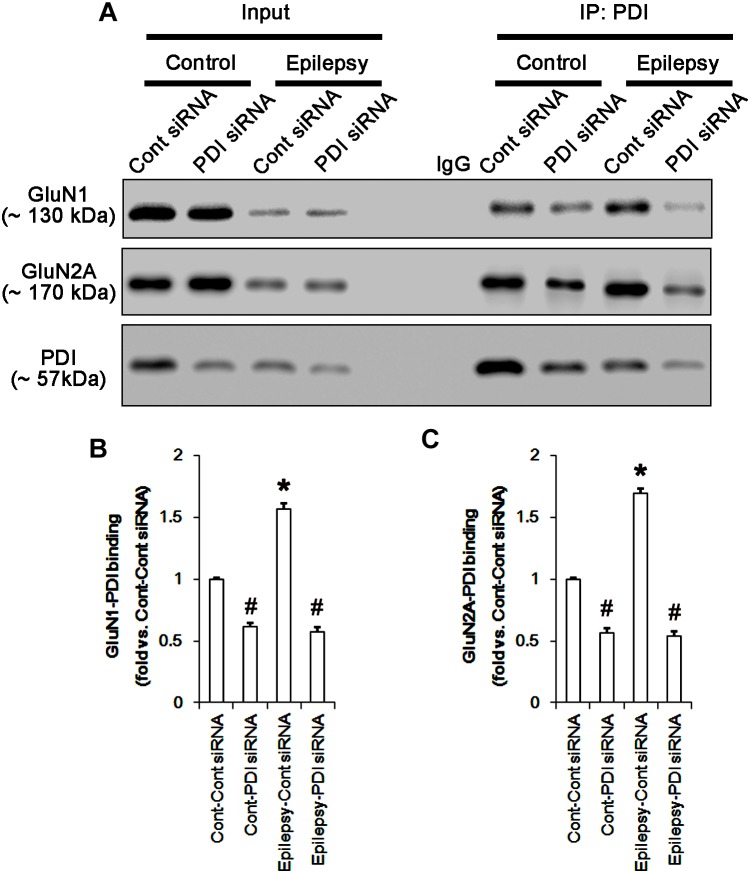
The effect of PDI knockdown on the binding of PDI to NMDAR subunits in chronic epilepsy model. **(A)** Co-immunoprecipitation of PDI and NMDAR. PDI siRNA reduce the PDI-NMDAR bindings in control and epilepsy animals. **(B,C)** Quantitative analyses of co-immunoprecipitation of PDI with GluN1 and GluN2A. Error bars indicate SEM (*^∗^,^#^p* < 0.05 vs. control; *n* = 7, respectively).

As compared to control siRNA, PDI knockdown did not affect expression levels and *S*-nitrosylation of NMDAR subunits. However, it decreased the amount of total thiols on GluN1 and GluN2A in chronic epilepsy rats (*p* < 0.05 vs. control siRNA; Figures 12A–C and Supplementary Figure 10). Thus, PDI knockdown increased the SNO-thiol-to-total thiol ratio on GluN1 and GluN2A to 1.57- and 1.45-fold of control siRNA-infused control animals, respectively (*p* < 0.05; Figures 12B,C). PDI siRNA reduced PDI expression without altering the amount of total thiol, *S*-nitrosylation and the SNO-thiol-to-total thiol ratio on PDI (*p* < 0.05 vs. control siRNA; Figures 12A3,D). PDI knockdown also alleviated the bindings of PDI to GluN1 and GluN2A (*p* < 0.05 vs. control siRNA; Figures 13A–C and Supplementary Figure 11). Taken together, our findings suggest that that PDI may not be a NO donor or a denitrosylase for NMDAR subunits, and that increase in total thiol levels on NMDAR by PDI siRNA may inhibit spontaneous seizure activity in epilepsy rats.

### PDI Knockdown Reduces Neuronal Activity in Response to NMDA

**FIGURE 14:**
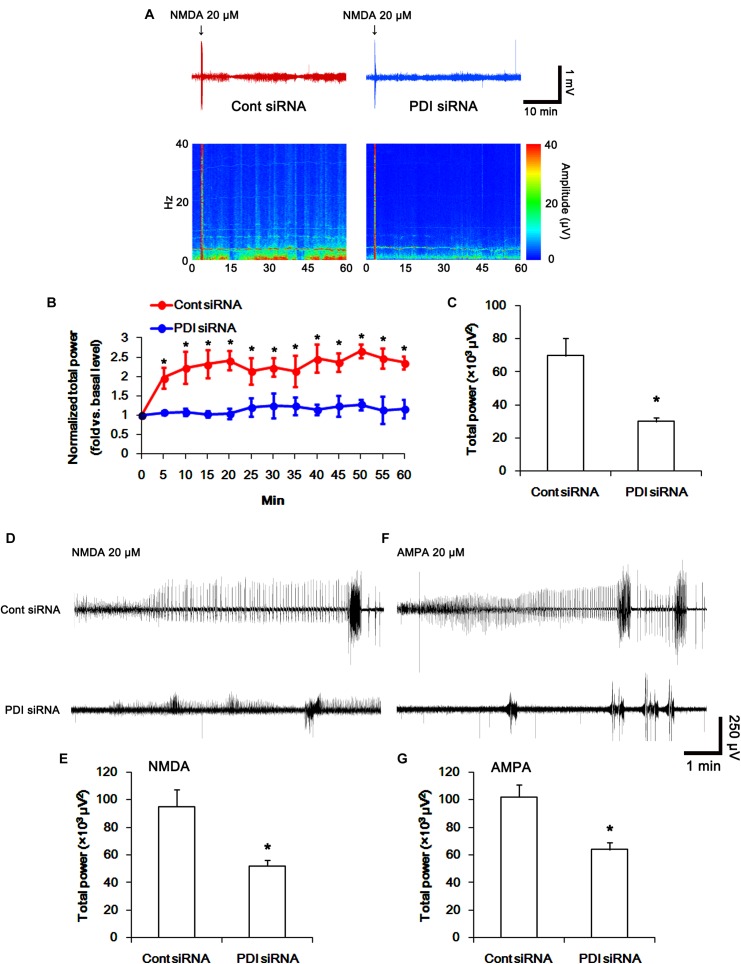
The effect of PDI siRNA on neuronal activity in response to NMDA and AMPA in control animals. PDI knockdown effectively inhibits neuronal excitation in response to NMDA. **(A)** Representative EEG trace and frequency-power spectral temporal maps in response to intracerebroventricular NMDA injection. **(B)** Quantification of normalized total EEG power in response to NMDA (mean ± S.E.M.; *^∗^p* < 0.05 vs. basal level; *n* = 7, respectively). **(C)** Quantification of total EEG power in response to NMDA (mean ± S.E.M.; *^∗^p* < 0.05 vs. control siRNA; *n* = 7, respectively). **(D)** Representative EEG trace in response to focal NMDA injection into the hippocampus. **(E)** Quantification of total EEG power in response to focal NMDA injection into the hippocampus (mean ± S.E.M.; *^∗^p* < 0.05 vs. basal level; *n* = 7, respectively). **(F)** Representative EEG trace in response to focal AMPA injection into the hippocampus. **(G)** Quantification of total EEG power in response to focal AMPA injection into the hippocampus (mean ± S.E.M.; *^∗^p* < 0.05 vs. basal level; *n* = 7, respectively).

To directly confirm PDI-mediated regulation of NMDAR activity, we applied NMDA injection (20 μM) into the ventricle. NMDA increased the amplitude, frequency of neuronal discharges and EEG total power in control siRNA-treated animals (*p* < 0.05 vs. basal level; *n* = 7; Figures 14A–C). However, NMDA did not affect them in PDI siRNA-treated rats, as compared the basal level (*p* < 0.05 vs. control siRNA; *n* = 7; Figures 14A–C). Similarly, the direct infusion of NMDA and AMPA into the hippocampus evoked epileptiform discharges in control siRNA-treated animals, while PDI knockdown inhibited field potentials in response to the direct NMDA or AMPA infusion (*p* < 0.05 vs. control siRNA; *n* = 7; Figures 14D–G). These findings indicate that PDI siRNA may reduce NMDAR functionality via regulating thiolation of NMDAR subunits.

## Discussion

The major findings in the present study are that PDI was not a NO donor or a denitrosylase for NMDAR, and that PDI knockdown inhibited seizure activity in acute seizure and spontaneous seizure activity in chronic epilepsy rats, independent of *S*-nitrosylation on NMDAR.

NMDAR over-activation results in neuronal death in a variety of acute and chronic neurological diseases including epilepsy (Lipton and Rosenberg, 1994). Thus, the maintenance of appropriate NMDAR activity is one of the potential antiepileptic and neuroprotective strategies. However, therapeutic doses of NMDAR antagonists result in the severe adverse effects including cognitive defects (Kornhuber and Weller, 1997; Chapman, 1998; Dannhardt and Kohl, 1998). The cysteine residues on NMDAR are involved in redox modulation and *S*-nitrosylation (Sucher and Lipton, 1991; Sucher et al., 1996; Lipton et al., 2002), which regulate NMDAR-mediate currents (Choi et al., 2000, 2001). Therefore, it is likely that the modulation of these post-translational modifications may be an interesting therapeutic target against epilepsy. Indeed, we have reported that PDI siRNA decreases seizure susceptibility by inhibiting the direct thiol reductase activity of PDI on NMDAR without glutathionylation of thiol modification in acute seizure- and chronic epilepsy model (Kim et al., 2017c). Since *S*-nitrosylation is a redox-based post-translational modification (Lei et al., 1992; Choi et al., 2000; Lipton et al., 2002), it is expected that PDI-mediated thiolation would affect the *S*-nitrosylation on NMDAR, but it has not been elucidated.

In the present study, acute seizure- and chronic epilepsy models showed the reductions in GluN1 and GluN2A expression levels. These findings are consistent with previous studies demonstrating the reduced NMDAR expression in acute seizure- and epilepsy rats (Lasón et al., 1997; Suh et al., 2001; Khan et al., 2008). Since NMDAR is inactivated by intracellular Ca^2+^ through the *C*-terminal splicing of the GluN1 subunit (Legendre et al., 1993; Okabe et al., 1999), it is likely that the reduced NMDAR expression may be a compensatory reaction to regulate the seizure activity by diminishing NMDA receptor responsiveness. Adversely, it could not be excluded that the diminished NMDAR expression may be due to massive neuronal loss in these models. In the present study, both acute seizure- and chronic epilepsy models showed the elevations of SNO-thiol levels on NMDAR subunit and PDI-NMDAR subunit bindings. PDI is a modulator of *S*-nitrosylation, since it regulates the entry of SNO-thiols into cells (Ramachandran et al., 2001) and denitrosylates SNO-thiols (Root et al., 2004). PDI itself also acts as a NO carrier by the formation of SNO-PDI (Sliskovic et al., 2005; Kallakunta et al., 2013). Therefore, it is likely that PDI would also regulate NMDAR functionality via *S*-nitrosylation in both animal models. However, PDI siRNA effectively inhibited seizure activities in acute seizure- and epilepsy animals without changing *S*-nitrosylation levels of NMDAR. Furthermore, PDI siRNA reduced the amplitude and frequency of neuronal discharges induced by intracerebroventricular and intrahippocampal NMDA and AMPA injection in control animals. The present data also reveal that PDI siRNA increased the fraction of the amount of SNO-thiols in total thiols on NMDAR subunits due to diminishing total thiol levels. Unlike PDI knockdown, L-NAME could not influence seizure activities in both models, although it reduced SNO-thiol, not total thiol, level on NMDAR. Therefore, these findings indicate that PDI may not be a NO donor or a denitrosylase for NMDAR, and that PDI-mediated reduction of disulfide bonds on NMDAR rather than *S*-nitrosylation on this receptor may relevant to seizure generations. Since NMDAR activation regulates conductance of the AMPA receptor (AMPAR; Selvakumar et al., 2013), furthermore, our findings suggest that the PDI siRNA may affect AMPAR activity by inhibiting NMDAR functionality.

Protein disulfide isomerase is originally a chaperone in the ER (Edman et al., 1985; Koch, 1987; Yoshimori et al., 1990). Therefore, it is likely that PDI siRNA would lead to ER stress. Consistent with our previous study (Kim et al., 2017c), however, the present data reveal that PDI siRNA did not affect the phosphorylation or expression levels of PERK, IRE1α, ATF6 and GRP78, which are indicatives of ER stress (Ko et al., 2015b; Kim et al., 2017c, 2018). Furthermore, SE induces ER stress in astrocytes rather than neurons, which provoke autophagic astroglial death (clasmatodendrosis; Ko et al., 2015b; Kim et al., 2017a, 2018). Therefore, our findings suggest that PDI knockdown may not induce ER stress in neurons under physiological condition.

On the other hand, *S*-nitrosylation of the active-site thiols of PDI inhibits its isomerase activity (Uehara et al., 2006). Therefore, it is plausible that the diminished PDI activity by *S*-nitrosylation would also affect its isomerase or reductase activity in both animal models. However, the present study shows that L-NAME could not affect the total thiol levels on NMDAR and PDI bindings to GluN1 and GluN2A in both models, although it reduced SNO-PDI level. Furthermore, PDI siRNA effectively decreased total thiol levels on NMDAR without changing the degree of *S*-nitrosylation of PDI. These findings indicate that *S*-nitrosylation of PDI may not influence its reductase activity and the PDI-NMDAR bindings at least in seizure or epilepsy models.

There are many debates concerning the role of NO in seizure activity. Some investigators claim that NO is one of the endogenous anti-convulsive factors, since L-NAME or other NOS inhibitors show proconvulsive effects against pilocarpine- and NMDA-induced seizure activity (Starr and Starr, 1993; Przegaliñski et al., 1996; Riazi et al., 2006; Capannolo et al., 2014) However, others reported that NO has proconvulsive properties (Vasconcelos Rios et al., 2013; Payandemehr et al., 2015). Moreover, NO is neither proconvulsive nor anti-convulsive substance for chronic spontaneous seizures, pilocarpine-, petylenetetrazol- or electroshock-induced acute seizures (Przegaliñski et al., 1996; Noyan et al., 2007). In the present study, L-NAME could not abrogate seizure activities in both animal models. Therefore, our findings indicate that NO synthesis may be a consequent response to seizure activity, and that NO itself may not be directly involved in ictogenesis. The present study also reveals that seizure on-set increased NO generation, which maintained after seizure termination in the acute seizure model. In contrast, NO level immediately elevated after spontaneous seizure on-set, and recovered to basal level after seizure cessation in chronic epilepsy animals. We cannot explain these differences in NO synthesis between two animal models in the present study. However, PDI siRNA-treated animals showed the shorter seizure duration in response to pilocarpine and the reverse of NO signal to basal level following diazepam. Therefore, it is plausible that the different seizure duration in acute seizure model and chronic epilepsy model would result in these discrepancies of NO generation. Conversely, it is also possible that the basal level of NO synthesis in epilepsy rats would be higher than that in control animals that used as acute seizure models. Further studies are needed to elucidate the underlying mechanisms of these discrepancies.

## Conclusion

In conclusion, our present data demonstrate that PDI was not a NO donor or a denitrosylase for NMDAR, and that PDI siRNA inhibited seizure activity by the *S*-nitrosylation-independent reduction of disulfide bonds on NMDAR. Therefore, our findings suggest that the modification of PDI reductase activity may be one of the important factors for the regulation of neuronal activity and the potential anti-epileptic therapeutic strategies.

## Author Contributions

J-EK designed and supervised the project. AJ designed and performed the experiments described in the manuscript with J-EK and analyzed the data. J-EK wrote the manuscript.

## Conflict of Interest Statement

The authors declare that the research was conducted in the absence of any commercial or financial relationships that could be construed as a potential conflict of interest.
