# Supplementary material for: PDI Knockdown Inhibits Seizure Activity in Acute Seizure and Chronic Epilepsy Rat Models via S-Nitrosylation-Independent Thiolation on NMDA Receptor
Source: Front Cell Neurosci. 2018 Nov 22;12:438. doi: 10.3389/fncel.2018.00438 (PMC6261974; doi:10.3389/fncel.2018.00438)
Supplement: Supplementary file 1 [file Data_Sheet_1.pdf]

Supplementary information

PDI regulates the *S*-nitrosylated thiol-to-total thiol ratio on NMDA receptor and seizure activity in acute- and chronic epilepsy rat models

A Ran Jeon[1], Ji-Eun Kim[1,*]

[1]Department of Anatomy and Neurobiology, Institute of Epilepsy Research, College of Medicine, Hallym University, Chuncheon 200-702, South Korea.

* Correspondence should be addressed to J-EK (e-mail: jieunkim@hallym.ac.kr)

Running title: PDI-mediated thiolation on NMDAR independent of *S*-nitrosylation

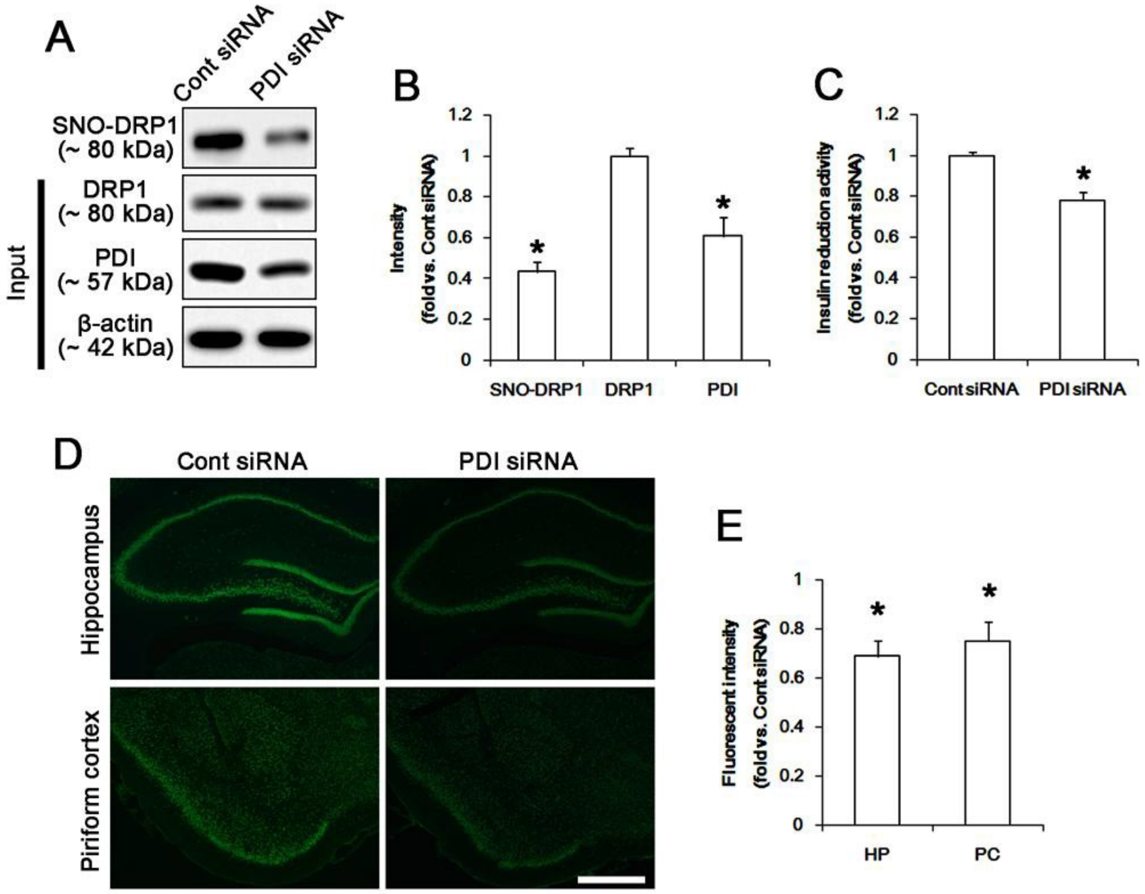

Supplementary Fig. 1. Efficacy of PDI siRNA on DRP1, PDI activity and its expression in the hippocampus. reduced PDI expression, its activity and *S*-nitrosylation of dynamin-related protein 1. **(A)** Representative western blot for SNO-DRP1, DRP1 and PDI. **(B)** Quantification of SNO-DRP1, DRP1 and PDI in response to PDI knockdown (mean ± S.E.M.; **p* < 0.05 vs. basal level; n = 7, respectively). **(C)** Quantification of PDI activity in response to PDI knockdown (mean ± S.E.M.; **p* < 0.05 vs. basal level; n = 7, respectively). **(D)** Representative images for PDI expression in the hippocampus and piriform cortex. Bar = 200 μm. **(E)** Quantification of PDI fluorescent intensity in response to PDI knockdown (mean ± S.E.M.; **p* < 0.05 vs. basal level; n = 7, respectively).

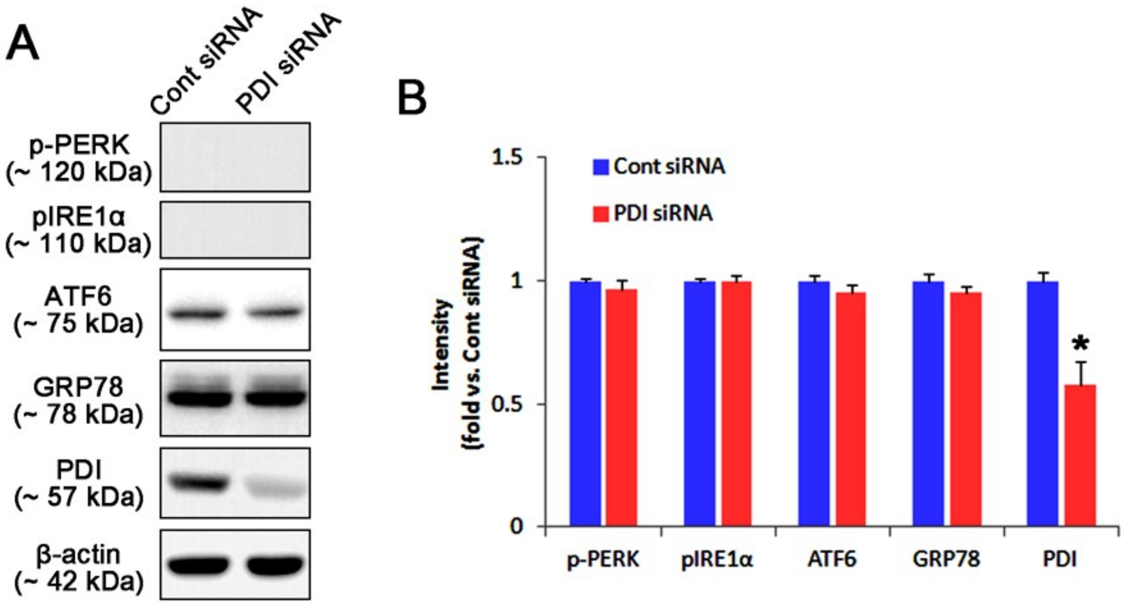

Supplementary Fig. 2. Effects of PDI siRNA on expression and phosphorylation of ER stress-related molecules. PDI knockdown does not provoke ER stress under physiological condition. **(A)** Representative western blot for expression and phosphorylation of ER stress-related molecules. **(B)** Quantification of expression and phosphorylation of ER stress-related molecules. Error bars indicate SEM (*,$^{\#}p < 0.05$ vs. control and vehicle, respectively; n = 7, respectively).

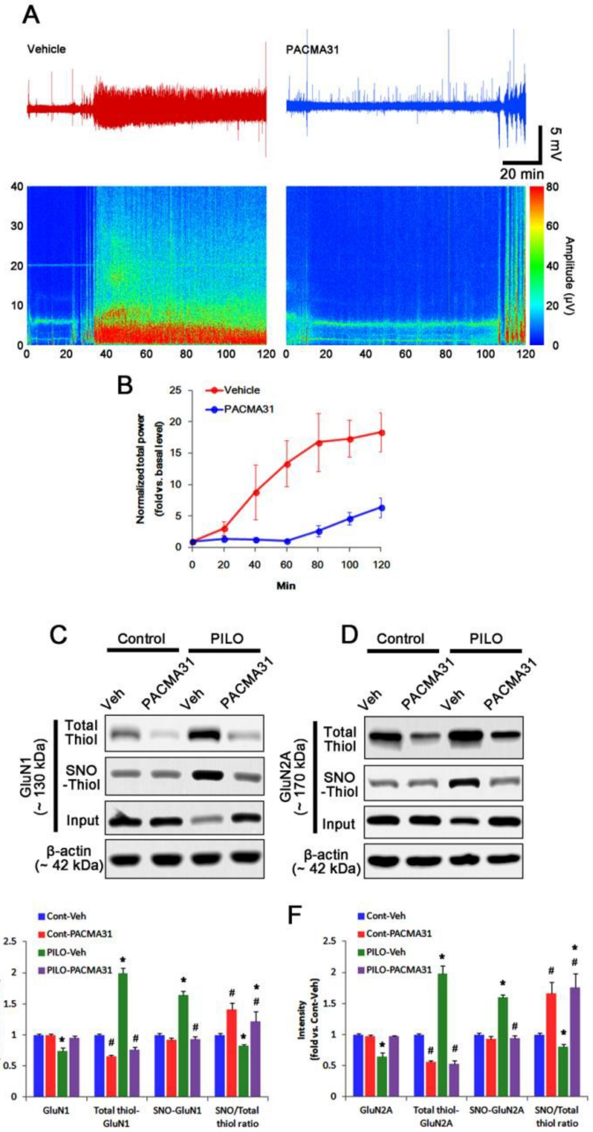

Supplementary Fig. 3. The effect of PACMA31 on acute seizure activity in response to pilocarpine. PACMA31 effectively inhibits seizure activity in response to pilocarpine. **(A)** Representative EEG trace and frequency-power spectral temporal maps in response to pilocarpine. **(B)** Quantification of total EEG power in response to PILO (mean ± S.E.M.; **p* < 0.05 vs. basal level; n = 7, respectively). **(C)** Representative western blot for expressions, and the amounts of total- and SNO-thiol on GluN1 and GluN2A. PACMA31 reduces SNO- and total thiol levels on both NMDAR subunits, but increases the SNO-thiol-to-total thiol ratios on both NMDAR subunits. **(E-F)** Quantification of expressions, the amounts of total thiols, SNO-thiol (panel 3) and the SNO-thiol-to-total thiol ratio on GluN1 (E) and GluN2A (F). Error bars indicate SEM ($*,^{\#}p < 0.05$ vs. control and vehicle, respectively; n = 7, respectively).

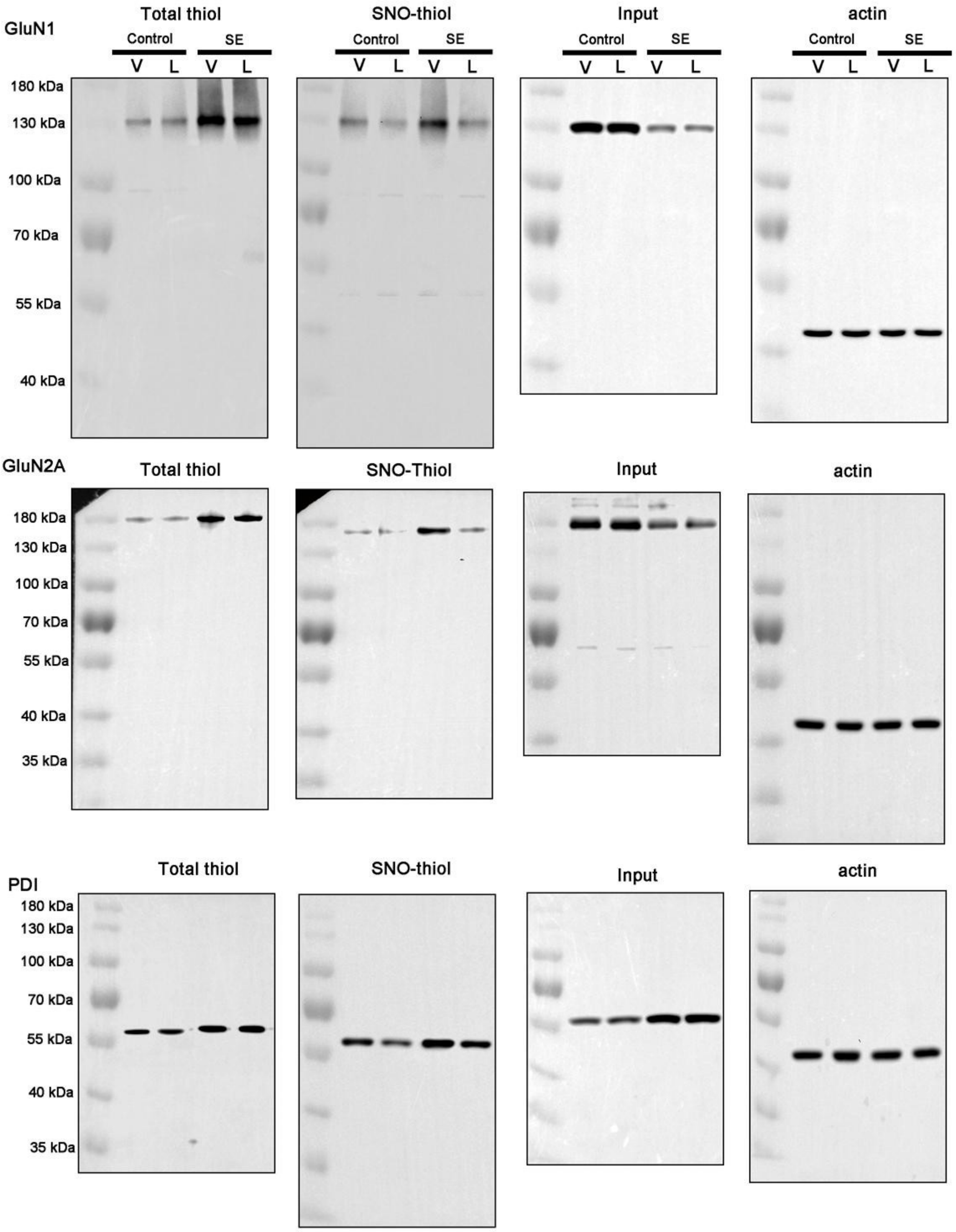

Supplementary Fig. 4. Full-length gel images of western blot data in Fig. 3. (V, vehicle; L, L-NAME).

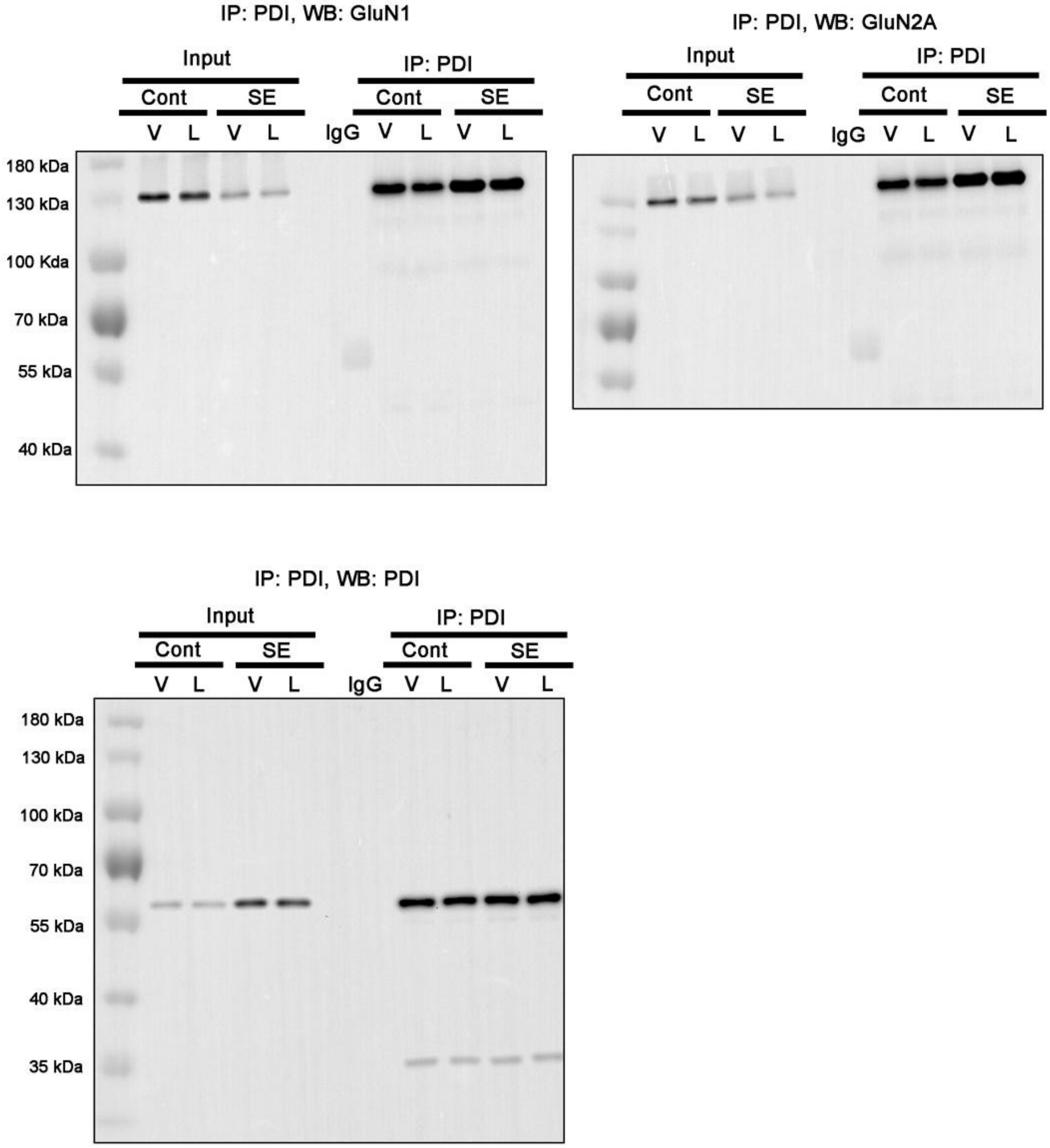

Supplementary Fig. 5. Full-length gel images of western blot data in Fig. 4. (V, vehicle; L, L-NAME).

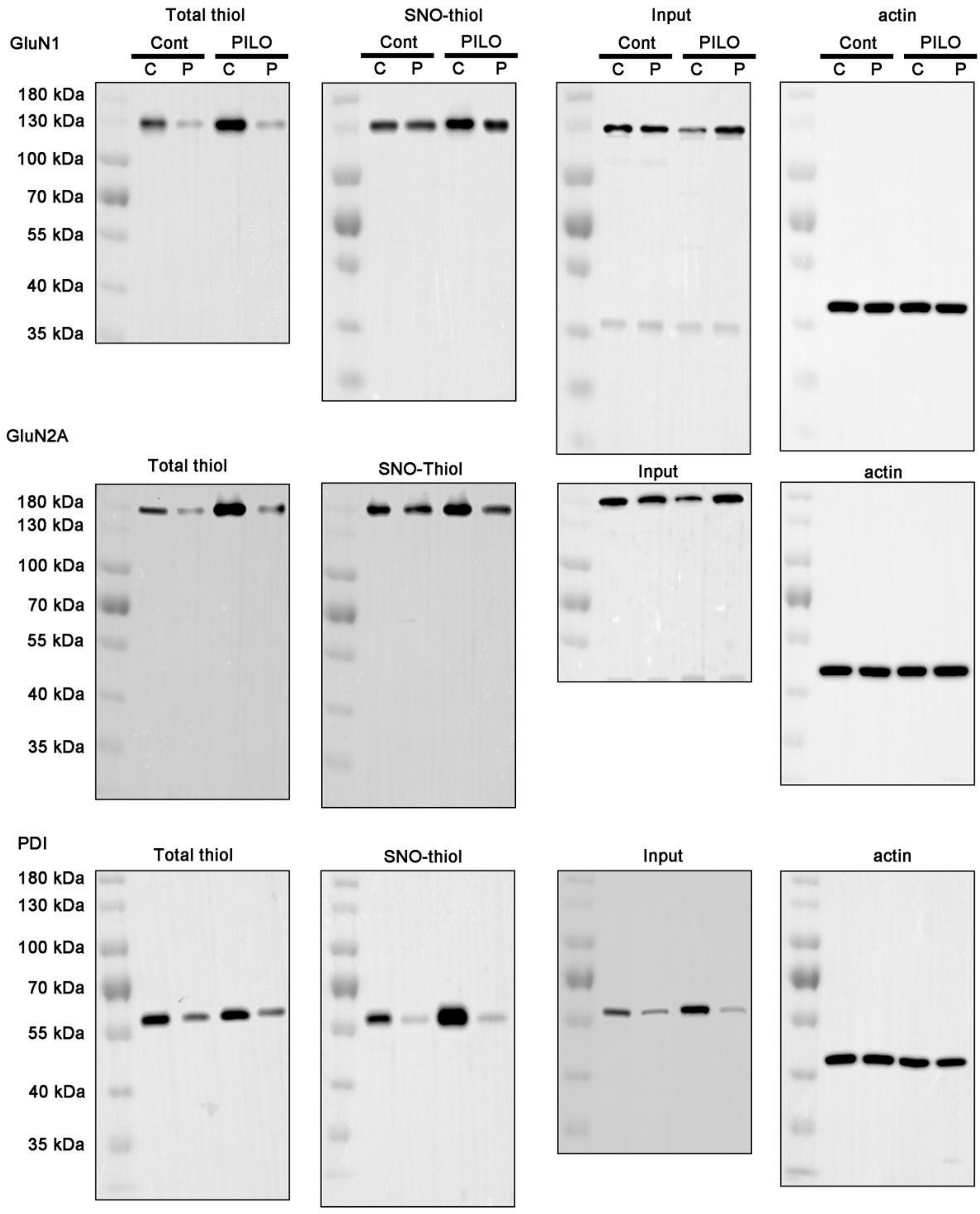

Supplementary Fig. 6. Full-length gel images of western blot data in Fig. 5. (C, control siRNA; P, PDI siRNA).

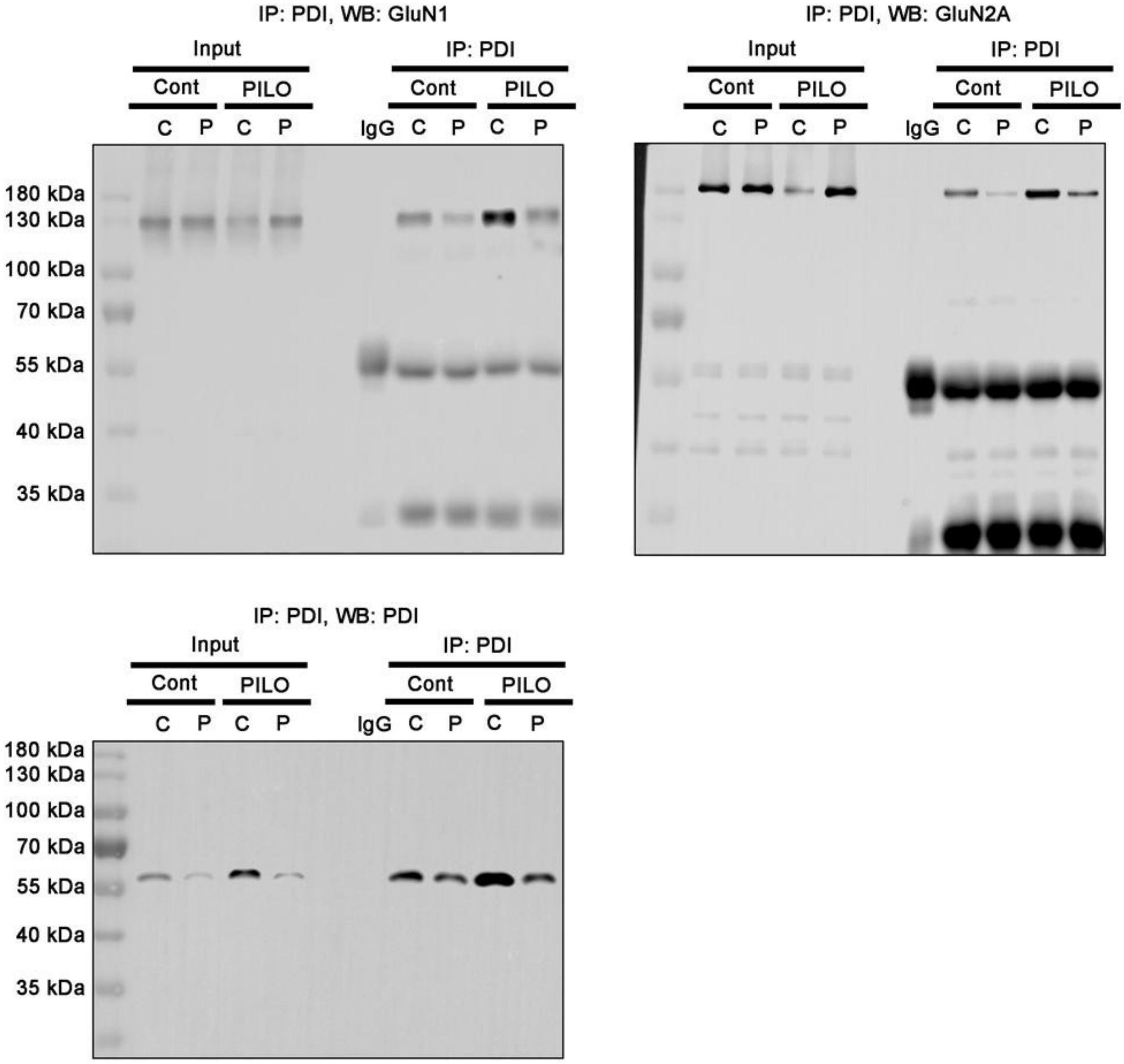

Supplementary Fig. 7. Full-length gel images of western blot data in Fig. 7. (C, control siRNA; P, PDI siRNA).

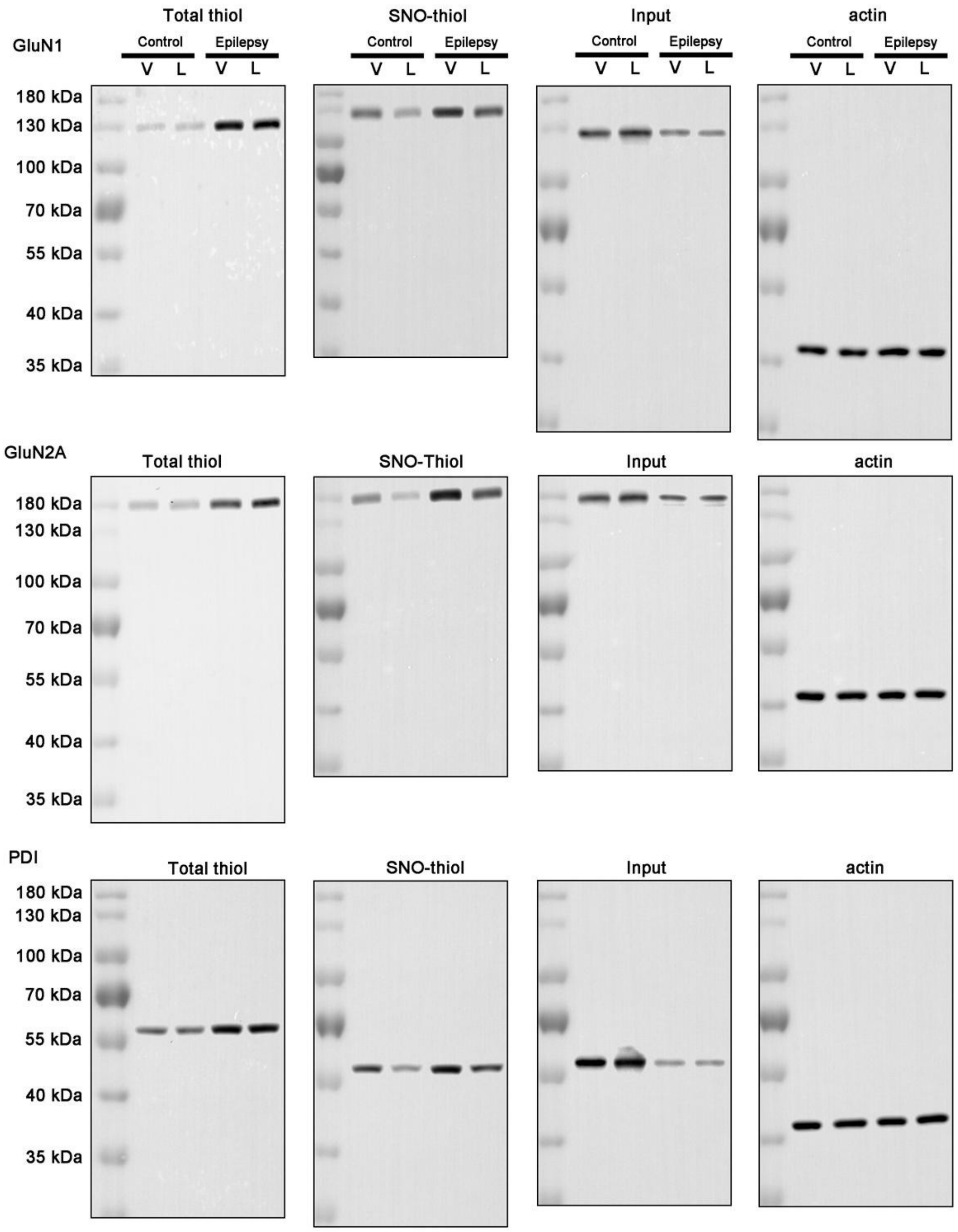

Supplementary Fig. 8. Full-length gel images of western blot data in Fig. 9. (V, vehicle; L, L-NAME).

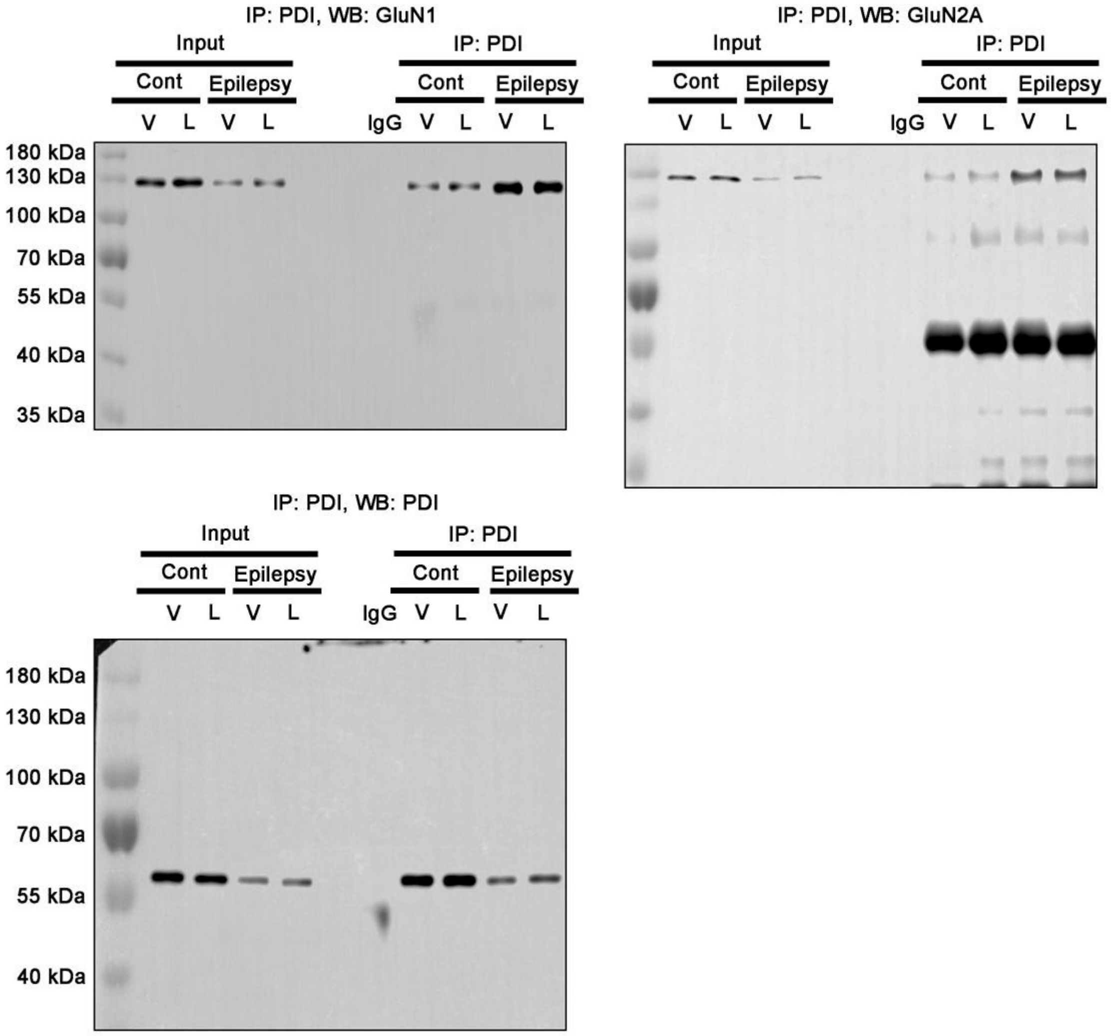

Supplementary Fig. 9. Full-length gel images of western blot data in Fig. 10. (V, vehicle; L, L-NAME).

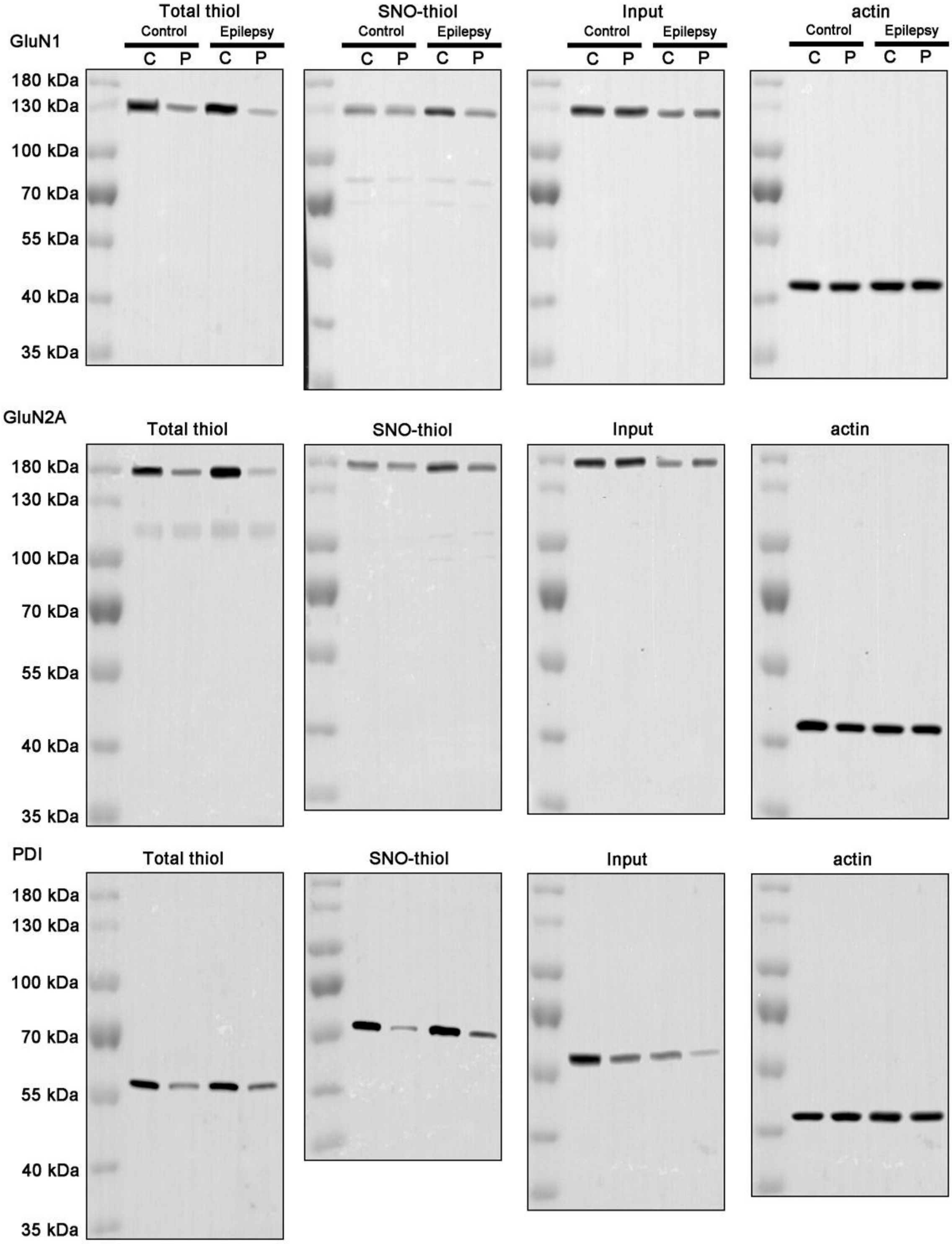

Supplementary Fig. 10. Full-length gel images of western blot data in Fig. 12. (C, control siRNA; P, PDI siRNA).

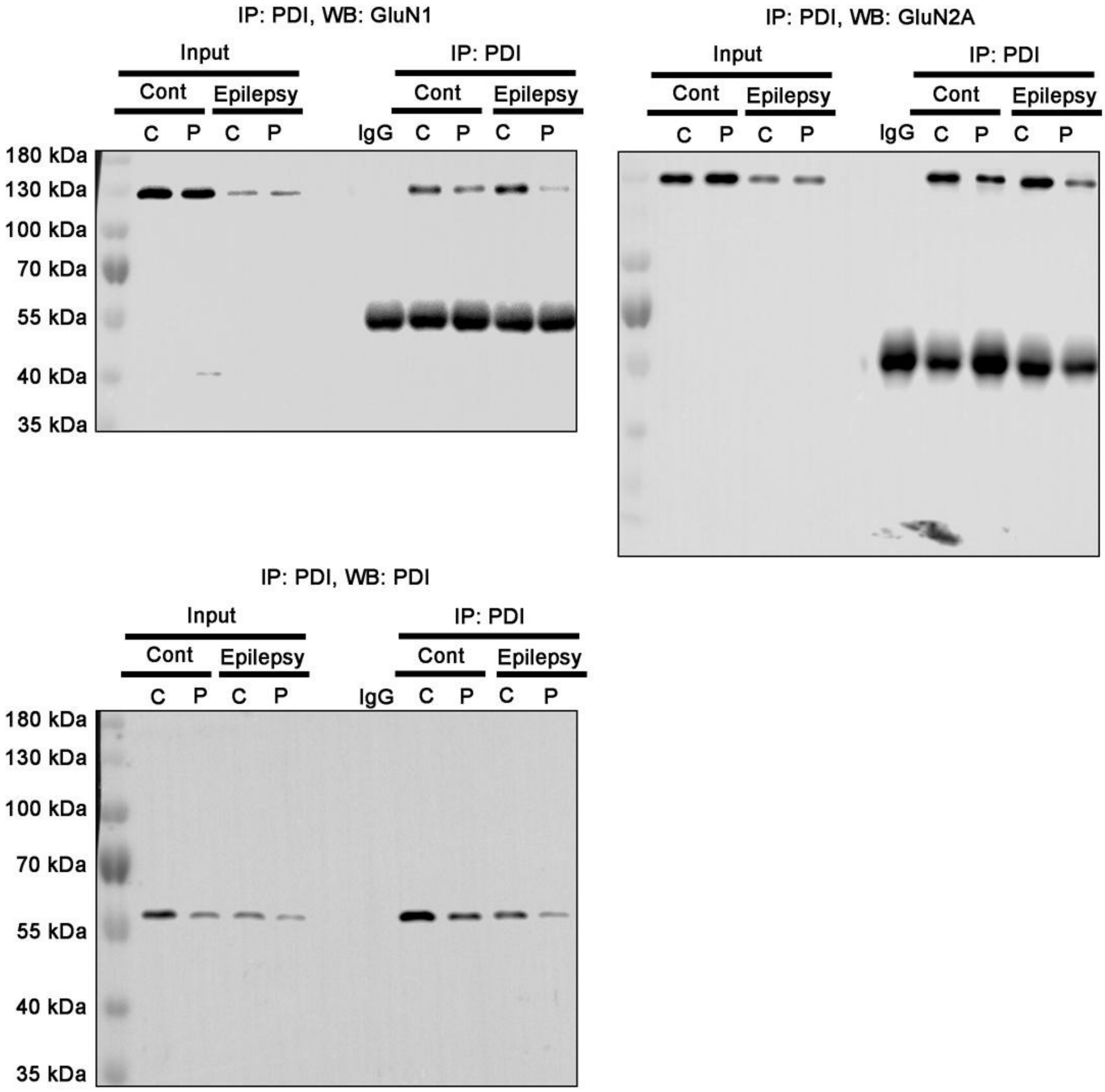

Supplementary Fig. 11. Full-length gel images of western blot data in Fig. 13. (C, control siRNA; P, PDI siRNA).

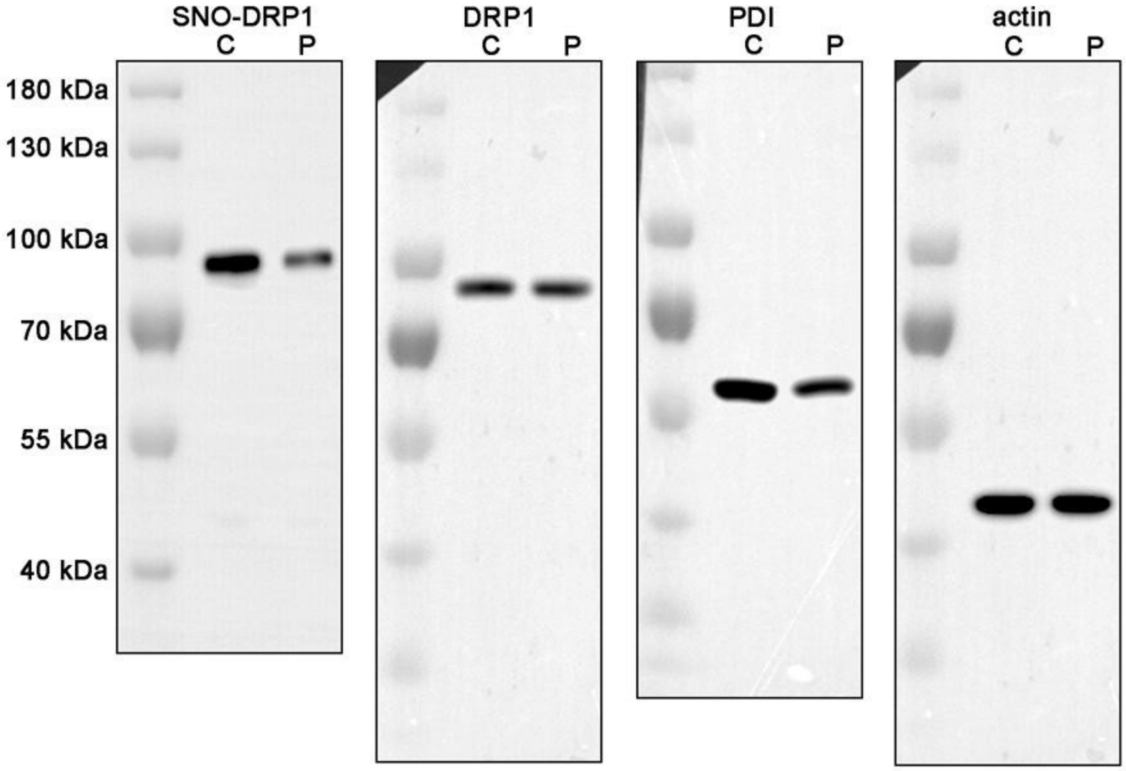

Supplementary Fig. 12. Full-length gel images of western blot data in Supplementary Figure 1. (C, control siRNA; P, PDI siRNA).

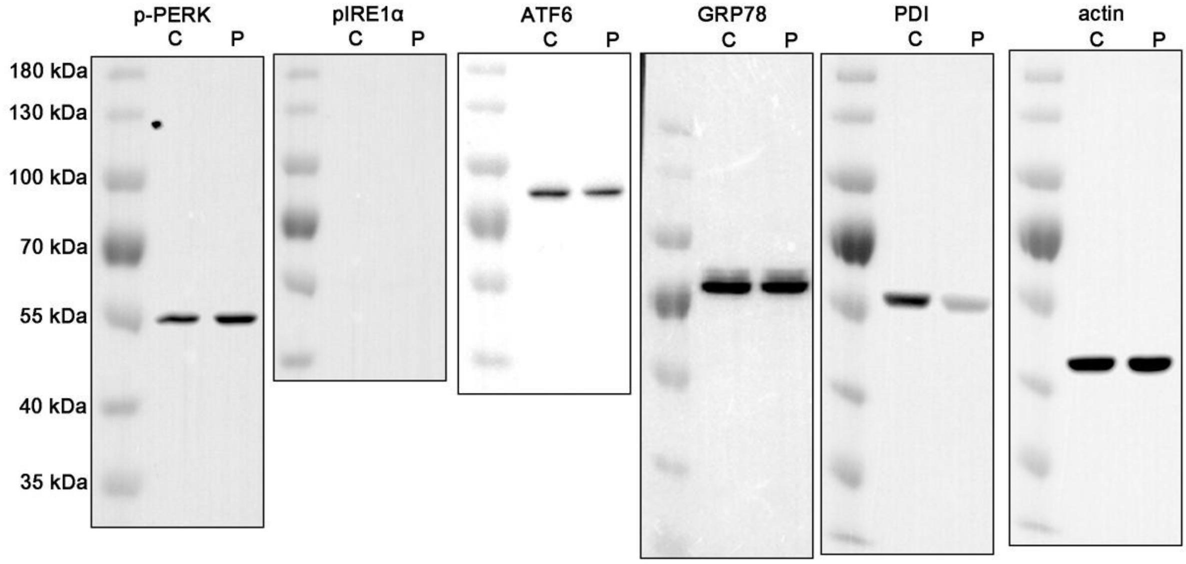

Supplementary Fig. 13. Full-length gel images of western blot data in Supplementary Figure 2. (C, control siRNA; P, PDI siRNA).

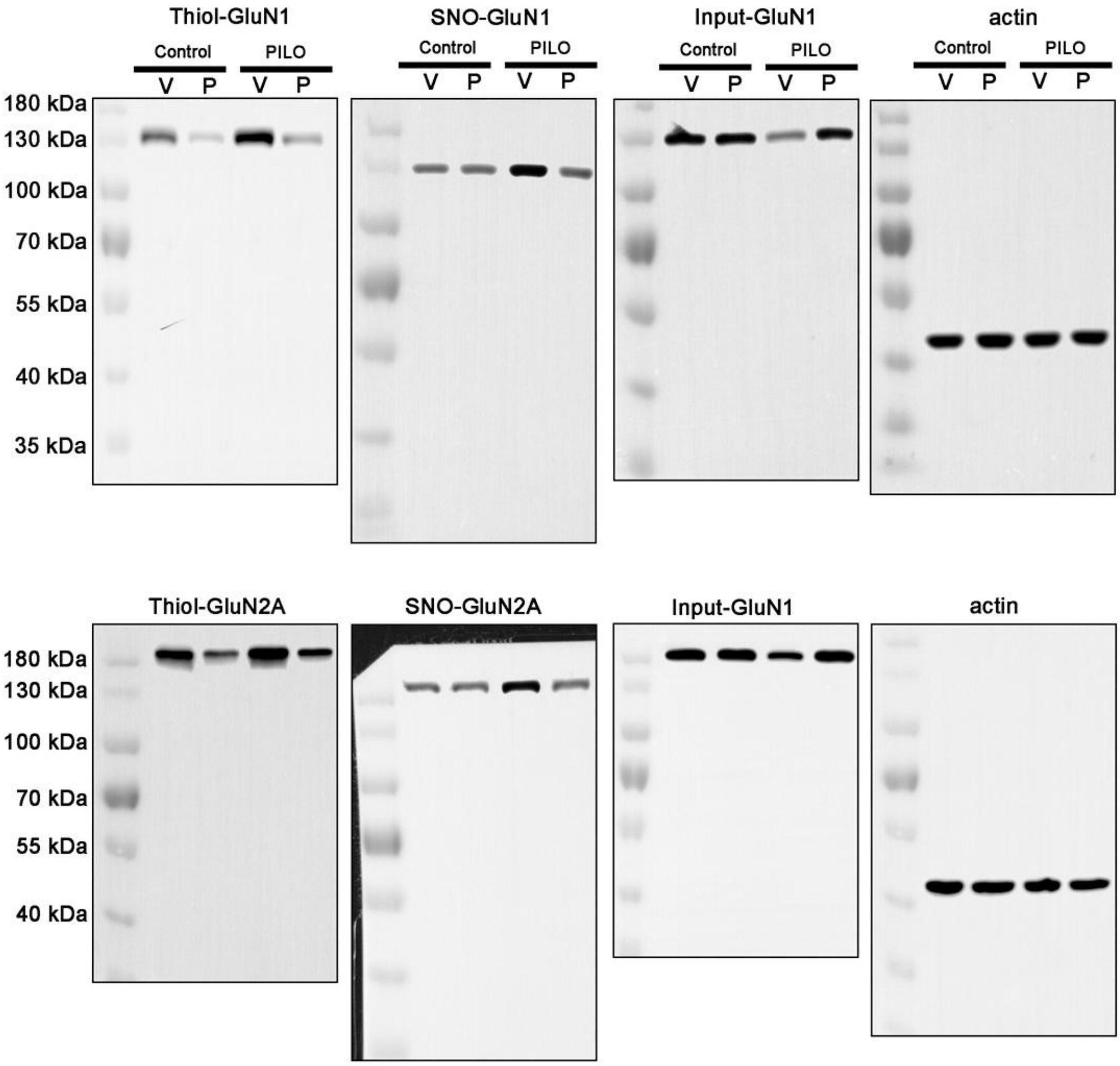

Supplementary Fig. 14. Full-length gel images of western blot data in Supplementary Figure 3. (V, vehicle; P, PACMA31).
